# FABP5 promotes lymph node metastasis in cervical cancer by reprogramming fatty acid metabolism

**DOI:** 10.7150/thno.44868

**Published:** 2020-05-17

**Authors:** Chunyu Zhang, Yuandong Liao, Pan Liu, Qiqiao Du, Yanchun Liang, Shiyin Ooi, Shuhang Qin, Shanyang He, Shuzhong Yao, Wei Wang

**Affiliations:** Department of Obstetrics and Gynecology, the First Affiliated Hospital, Sun Yat-sen University, Guangzhou 510080, Guangdong, China

**Keywords:** FABP5, lymph node metastasis, cervical cancer, fatty acid metabolism, NF-κB signaling pathway

## Abstract

Patients with cervical cancer (CCa) with lymph node metastasis (LNM) have an extremely poor prognosis. Elucidation of the molecular mechanisms underlying LNM may provide clinical therapeutic strategies for CCa. Upregulation of fatty acid-binding protein 5 (FABP5) expression in CCa tumours was demonstrated to positively correlate with LNM. However, the precise role and mechanisms of FABP5 in the LNM of CCa remain unknown.

**Methods**: The diagnostic value of FABP5 as a predictor of LNM in CCa was evaluated in CCa tumour samples. The functional role of FABP5 and its upstream and downstream regulatory factors were investigated by gain-of-function and loss-of-function assays in vitro and in vivo. A mouse model of LNM was used to determine the effect of FABP5 on LNM and the therapeutic value of FABP5 targeting.

**Results**: We demonstrated that FABP5 was markedly upregulated in CCa with LNM and correlated with poor prognosis. FABP5 protein was an independent predictor of LNM in a multivariate logistic analysis. Furthermore, FABP5 promoted epithelial-mesenchymal transition, lymphangiogenesis, and LNM by reprogramming fatty acid (FA) metabolism. Mechanistically, FABP5 promoted lipolysis and FA synthesis, which led to an increase in intracellular fatty acids (FAs) that activated NF-κB signalling, thus inducing LNM. Importantly, administration of orlistat, which attenuates FA metabolism reprogramming, inhibited FABP5-induced LNM in CCa. The pro-metastatic effect of FABP5 was reduced by miR-144-3p. Moreover, miR-144-3p was significantly downregulated and FABP5 was upregulated in CCa in a hypoxic microenvironment.

**Conclusion**: Our findings highlight a FA metabolism-dependent mechanism of FABP5-induced LNM. Moreover, the expression and biological function of FABP5 can be regulated by miR-144-3p in hypoxia. Our study identifies FABP5 as a potential diagnostic biomarker and therapeutic target for LNM in CCa.

## Introduction

Cervical cancer (CCa) is the second most common cancer in less developed countries 1. In China, there are estimated 98,900 new cases and 30,500 related deaths annually 2. The lymphatic system is the most common route of the spread of CCa, even in the early stage 3. The prognosis of patients with CCa is associated with the presence of lymph node metastasis (LNM), which decreases the 5-year survival rate from 95% to 33.3% 4. However, there is still no effective and reliable means to prevent or control LNM. Thus, investigations of the molecular mechanisms underlying LNM and the identification of novel, promising targets for the therapeutic intervention of LNM are urgently needed for patients with CCa.

The reprogramming of cellular energy metabolism has been increasingly recognized as a hallmark of cancer 5. The Warburg effect is the most common metabolic perturbation in carcinogenesis 6. However, the molecular mechanisms involved in carcinogenesis are different from those in cancer metastasis. Accumulating evidence demonstrates that the reprogramming of lipid metabolism plays a crucial role in tumour metastasis 7. Lipid metabolism reprogramming alters the production of many bioactive lipid molecules, such as fatty acids (FAs). It is widely appreciated that FAs are fundamental components of cancer cells because they are the main building blocks for membrane biosynthesis and provide fuel sources during conditions of metabolic stress. More importantly, alteration of fatty acid metabolism in cancer has received renewed interest as a key secondary messenger to create a microenvironment that contributes to tumour progression 8-12. However, it remains unknown how CCa cells remodel lipid metabolism, thus resulting in LNM.

We previously demonstrated that high expression of fatty acid-binding protein 5 (FABP5) is positively correlated with the presence of LNM of CCa 13, 14. FABP5 is a low molecular weight cytoplasmic protein and is a member of the fatty acid-binding protein (FABP) family. FABPs are demonstrated to be important regulators of FA metabolism 15. FABP5 has been reported to be upregulated in some cancers. Moreover, recent studies have shown that FABP5 participates in cancer progression 16-24. This evidence led us to speculate whether FABP5 promotes LNM through reprogramming of FA metabolism in CCa. However, the molecular mechanism underlying the relationship among FABP5, aberrant tumour FA metabolism and LNM in CCa remains elusive.

Herein, we report that FABP5 is an independent predictor of LNM and a valuable prognostic factor in CCa. FABP5 facilitated epithelial-to-mesenchymal transition (EMT), lymphangiogenesis, and LNM in vitro and in vivo. Mechanistically, FABP5 promotes lipolysis of lipid droplets and de novo fatty acid synthesis, leading to an increase in intracellular FAs. Then, excess intracellular FAs activate the NF-κB signalling pathway, resulting in LNM in CCa. We found that FABP5 was directly targeted and suppressed by miR-144-3p, which influences its expression and pro-metastatic effect. Moreover, the expression of FABP5 and miR-144-3p was regulated by hypoxia. Our findings highlight an FA metabolism-dependent mechanism of FABP5-induced LNM and identify FABP5 as a potential diagnostic marker and therapeutic target for LNM of CCa.

## Methods

### Patients and tissue specimens

All patients' specimens used in this study were conducted in accordance with the Declaration of Helsinki. All clinical specimens were collected after approval by the Ethical Committee of the First Affiliated Hospital of Sun Yat-sen University (Guangzhou, China). Informed consent was obtained from all participants or their appropriate surrogates. All specimens were handled according to legal and ethical standards. None of these patients received preoperative radiation or chemotherapy. For immunohistochemistry (IHC) analysis, 260 cervical squamous cell tumour samples (49 with LNM, 211 without LNM) were collected (Table S1). These paraffin-embedded CCa tumour tissues were collected from the archives of the pathology department at the First Affiliated Hospital of Sun Yat-sen University. Surgeries were performed between 2008 and 2018. Twenty normal uterine cervical tissue controls were collected from women who underwent hysterectomy for benign diseases at our centre between 2017 and 2018. Total RNA was isolated from 40 CCa tumour samples (20 with LNM, 20 without LNM) who underwent surgery at our centre from 2017 to 2018. Each tissue sample was assessed and dissected by an experienced pathologist immediately after tumour resection. The pathologist then confirmed that each dissected tumour tissue block was composed of at least 80% tumour cells by examining the frozen section. Tissues were immediately frozen in liquid nitrogen and then stored at -80℃ until further use.

### Cell culture

Human cervical cancer cell lines (SiHa, MS751, HeLa, ME180) were purchased from the American Type Culture Collection (ATCC). Siha, HeLa and ME180 cells were cultured in DMEM with 10% foetal bovine serum (FBS) (Gibco, USA). MS751 cells were cultured in MEM medium with 10% FBS. Human lymphatic endothelial cells (HLECs) were obtained from ScienCell Research Laboratories (Carlsbad, CA, USA) and cultured in the ECM (ScienCell, San Diego, CA, USA). Primary normal cervical epithelial cells (NCCs) were isolated from normal uterine cervical specimens as previously described 25.

### Cell transfection and plasmid construction

All the miRNA mimics and siRNAs of FABP5 were designed and synthesized by GenePharma (Suzhou, China) and were transfected into cancer cells using Lipofectamine RNAiMAX (Invitrogen, USA). The pENTER-His-FABP5 plasmid was synthesized by Vigene Biosciences (Shandong, China) and then transfected into cells using X-tremeGENE HP DNA transfection reagent (Roche, Mannheim, Germany). Lentivirus-packaged shFABP5 and FABP5 plasmids were purchased from Vigene Biosciences, and stably transfected cells were selected with puromycin (3 mg/ml, Sigma-Aldrich, USA) for 5 days. The siRNA and shRNA sequences are listed in Supplementary Table S1.

### IHC, qRT-PCR, Western blotting, and enzyme-linked immunosorbent assay (ELISA)

IHC, qRT-PCR, and Western blotting analysis were performed as previously described 25. The results of IHC were scored by adding the staining intensity (0 = no, 1 = weak, 2 = moderate, 3 = strong staining) and the staining area (0 = no, 1 = less than 30%, 2 = between 30% and 60%, 3 = between 60% and 100% stained cells). The immunostaining scores (ranging from 0 to 6) were evaluated by two experienced pathologists in a blinded fashion. For statistical analysis, the immunostaining scores were evaluated, and a cut-off was determined. The samples were divided accordingly into low- and high- staining groups. For FABP5, CPT1A, and ACC1, a staining score of 4 was defined as the cut-off. For FASN, ACOX1, CA9, and HSL, a staining score of 3 was defined as the cut-off. Nuclear/cytoplasmic fractionation of MS751 cells was performed by using the PARIS Kit (Invitrogen, USA) following the manufacturer's protocols. Antibodies and working concentrations are presented in Table S5. The primer sequences used in this study are provided in Table S4. ELISA was performed by using a vascular endothelial growth factor C (VEGF-C) ELISA kit (Raybio, Norcross, GA, USA) following the manufacturer's instructions.

### Cell proliferation assay, Transwell assay, and HLEC tube formation assay

Tumour cells were seeded into 96-well plates at a density of 5,000 cells/well. Then, CCK-8 solution (Dojindo, Japan) was carefully added to plates (10 μl/well). The plates were incubated at 37°C. The absorbance was detected at 450 nm by a microplate reader. For the Transwell assay, cells were seeded into the upper chamber precoated with 15% Matrigel (BD Biosciences, USA) with serum-free culture medium, while the lower chamber was filled with 500 μl complete medium. After 48 h, the cells on the lower surface of the chamber were fixed and then stained. The numbers of migrated cells were counted under a microscope. For the HLEC tube formation assay, HLECs were seeded into 48-well plates (precoated with Matrigel) containing cell culture medium and incubated for 10 h. Tube formation was quantified by measuring the total length of tube structures or the number of branch sites/nodes in 3 random fields.

### Quantification of metabolites

Quantification of fatty acids (FAs) was performed by using an EnzyChrom Free Fatty Acid Assay Kit (BioAssay Systems). Quantification of glycerol was performed using the Glycerol Cell-Based Assay Kit (Cayman Chemical, Ann Arbor, MI). Quantification of intracellular triacylglycerol (TAG), diacylglycerol (DAG), and monoacylglycerol (MAG) was performed by using the Adipogenesis Colorimetric/Fluorometric Assay Kit (Bio-vision, Milpitas, CA, USA). All assays were performed following the manufacturer's instructions.

### Nile red staining and Oil Red O staining

For cell lipid droplet quantification, Nile red staining was used. Transfected CCa cells were seeded in 6-well plates in which a sterile glass coverslip had been placed in advance. When the cell layer was between 60% and 80% confluent, the cells were fixed in 2 ml 2% formaldehyde in PBS for 20 min. The cells were then incubated with 1 ml Nile red staining solution (Sigma-Aldrich, USA) for 20 min and then stained with Hoechst 33342 (Beyotime, China) for 20 min at room temperature. The coverslips were mounted onto glass slides with 30 μl antifade mounting medium. For tissue lipid droplet quantification, Oil Red O (Sigma-Aldrich, USA) staining was performed. In brief, frozen sections were fixed in formalin and rinsed with 60% isopropanol. The sections were then stained with freshly prepared Oil Red O working solution for 15 min and dipped in alum haematoxylin 5 times. All images were photographed and analysed by using a Leica DM6B microscope.

### Assessment of fatty acid β-oxidation (FAO) and fatty acid uptake assay

Evaluation of FA β-oxidation in cervical cancer cells was performed as previously described 26, 27. Briefly, cancer cells in 6-well plates (1.5 × 10^6^ cells/well) were cultured with 250 μM palmitate for 16 h followed by incubation with 125 μM [^14^C]-palmitate for 2 h. FAO acid-soluble metabolites and captured ^14^CO_2_ were examined with an L6500 scintillation counter (Beckman Coulter, Brea, CA). The activity of FA uptake was measured by the QBT Fatty Acid Uptake Kit (Molecular Devices, Sunnyvale, CA, USA) according to the manufacturer's protocol. The fluorescence intensity was detected by the PowerScan HT microplate reader.

### Luciferase reporter assay

A luciferase assay was performed as previously described 25. Briefly, 3 × 10^4^ cells were seeded in 24-well plates. After 24 h, the cells were cotransfected with a mixture of 5 ng Renilla luciferase reporter vectors, 100 ng luciferase reporter vectors (pNFκB-luc, pGL3, pGL3-FABP5-3'UTR, or pGL3-FABP5-3'UTR-mut) and other transfection components (miR-144-3p mimics, pFABP5 or siRNAs). Luciferase and Renilla signals were measured 36 h after transfection using the Dual Luciferase Reporter Assay Kit (Promega, Madison, WI, USA) following the manufacturer's instructions.

### Xenograft model

All animal studies were conducted with the approval of the Animal Ethical and Welfare Committee of Sun Yat-sen University. Xenograft models were established as previously described 25. Female BALB/c nude mice were purchased from the Laboratory Animal Center of Sun Yat-sen University. For the subcutaneous tumorigenicity assay, 1×10^7^ cells stably overexpressing or silencing FABP5 in 200 μl PBS were inoculated subcutaneously. After injection, tumour size was measured by using an external caliper. For the xenograft LNM model, 1 × 10^7^ cells were inoculated into the footpads of the mice. The lymph node volumes were calculated using the following formula: volume (mm^3^) = (length [mm]) × (width [mm])^2^ × 0.52. The footpad tumours and popliteal lymph nodes were removed for haematoxylin and eosin (HE) staining and/or Oil Red O staining. For survival analysis, the mice were observed until they died or were sacrificed by cervical dislocation 60 days after the first injection of tumour cells. For orlistat administration, the mice were intraperitoneally injected with orlistat (240 mg/kg/d, Xenical^®^, F. Hoffman-La Roche, Basel, Switzerland) or the equivalent amount of vehicle (33% ethanol in PBS) for 25 days 28-30. To make a bevacizumab-treated tumour model, mice bearing tumours approximately 5 mm in diameter were used for subsequent experiments. In the experimental group, bevacizumab (Avastin^®^, Roche Pharma, Germany) (5 mg/kg twice a week) was administered for 3 weeks by intraperitoneal injection 15, 31.

### Statistical analyses

Statistical analyses were performed using SPSS 13.0 (SPSS Inc., Chicago, IL, USA), MedCalc statistical software (MedCalc Software, Mariakerke, Belgium), and Prism 5.0 software (GraphPad). The independent sample *t-*test was used to compare the difference between two groups. The relationships between FABP5 expression and clinicopathological characteristics were evaluated by the Pearson χ^2^ test or Fisher's exact test. Correlations between measured variables were analysed by Spearman rank correlation analysis. Multivariate logistic regression was performed to identify the independent predictor related to LNM of CCa. A multivariate Cox proportional hazard model analysis was used to identify independent prognostic factors of CCa. The Kaplan-Meier method was used for recurrence-free survival (RFS) and overall survival (OS) analysis, and the curves were compared by the log-rank test. *P* < 0.05 was considered to be statistically significant.

## Results

### FABP5 is correlated with LNM and predicts poor prognosis in CCa

**Figure 1:**
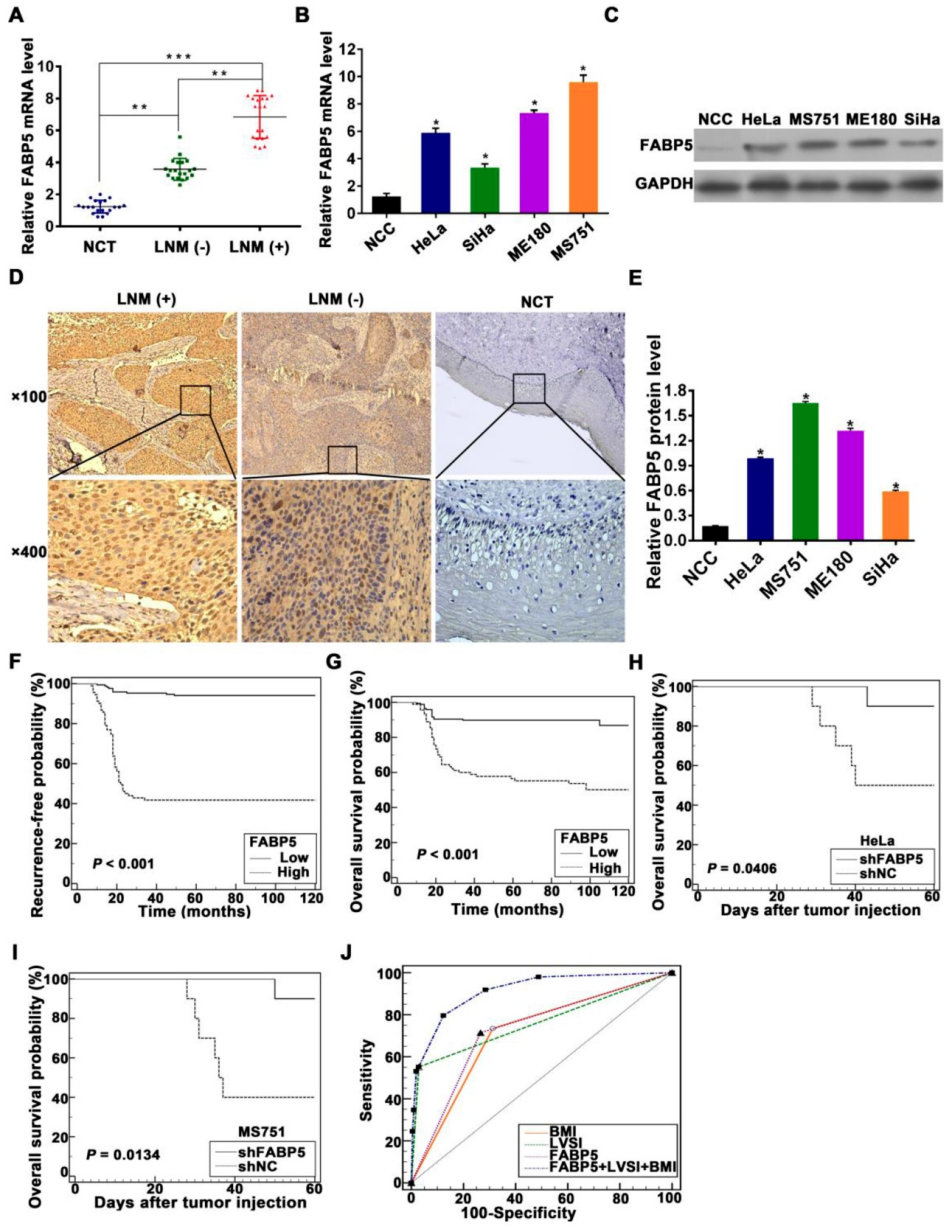
** FABP5 is associated with LNM and poor prognosis of CCa.** (A) The qRT-PCR results indicated that FABP5 was more highly expressed in the primary tumours of CCa with LNM than in those without LNM or normal uterine cervical tissues (NCTs) (n = 20 per group). **P < 0.01 and ***P < 0.001. (B) FABP5 mRNA expression in four CCa cell lines and normal cervical epithelial cells (NCCs). *P < 0.05. (C) Western blot assay of FABP5 protein expression in CCa cell lines and NCCs. (D) Representative results of IHC staining of FABP5 in CCa and NCT. (E) Bars represent relative protein quantification of FABP5 in CCa cell lines and NCCs. (F, G) Kaplan-Meier survival curves of RFS and OS of patients with CCa according to FABP5 expression. (H, I) Kaplan-Meier survival plots for mice injected with HeLa or MS751 cells into the foot pad (n = 10 per group). (J) ROC curves of FABP5, BMI, and LVSI as markers to predict LNM in CCa. Error bars represent the mean ± S.D. of three independent experiments.

To validate whether FABP5 plays a role in LNM, we first analysed the expression of FABP5 mRNA and protein in CCa tumours. FABP5 expression in primary CCa tumours with LNM was significantly higher than that in tumours without LNM and normal uterine cervical tissues (NCTs) at both mRNA and protein levels **(Figure 1A, 1D)**. We then investigated FABP5 mRNA and protein expression in CCa cell lines and normal cervical epithelial cells. The expression levels of FABP5 mRNA and protein were significantly higher in CCa cells than in normal cervical epithelial cells **(Figure 1B, 1C, 1E)**. Moreover, the expression levels of FABP5 mRNA and protein in CCa cells from lymph node metastatic sites (MS751) were higher than that in CCa cells derived from primary sites (HeLa and SiHa) **(Figure 1B, 1C, 1E)**.

To assess the association between FABP5 protein expression and clinicopathological factors in CCa, the expression of FABP5 protein was detected by IHC analysis of 260 paraffin-embedded CCa tumours. Our data showed that FABP5 protein expression was significantly higher in CCa tumours with LNM than in those without LNM and in NCTs **(Figure 1D)**. Moreover, FABP5 overexpression was significantly correlated with LNM (*P* = 0.001), body mass index (BMI) (*P* = 0.013), FIGO stage (*P* < 0.001), lymphovascular space invasion (LVSI) (*P* = 0.004), and tumour size (*P* = 0.02) **(Table S2)**. In multivariate Cox proportional hazard model analysis, age, LNM, tumour size, and FABP5 were found to be independent prognostic factors **(Table S3)**. Kaplan-Meier survival curves and log-rank test analyses indicated that patients with high FABP5 protein expression was related to significantly decreased RFS and OS **(Figure 1F-G)**. Moreover, in a nude mouse model of LNM, the group of mice harbouring stable FABP5-knockdown (shFABP5) cells exhibited improved survival compared to that of the control mice **(Figure 1H-I)**.

To determine whether FABP5 is a high-risk factor for LNM, a multivariate logistic regression analysis was constructed and showed that FABP5, LVSI, and BMI were independent predictors of LNM in CCa patients **(Table 1)**. Furthermore, to assess the discriminatory power of FABP5, BMI, and LVSI to predict LNM in CCa, receiver operating characteristic (ROC) curves were constructed **(Figure 1J)**. FABP5, BMI, and LVSI were used as covariates to create a logistic regression model with LNM as the dependent variable to further evaluate the feasibility of the combination of FABP5, BMI, and LVSI to predict LNM. The area under the ROC curve (AUC), sensitivity, specificity, positive predictive value (PPV), and negative predictive value (NPV) of FABP5, LVSI, BMI, and the combination of the three factors in the prediction of LNM are shown in Table 2. When LVSI, BMI, and FABP5 were combined, the discriminatory power for predicting the presence of LNM in CCa was strong (AUC = 0.913) **(Table 2)**. Moreover, the combination of LVSI, BMI, and FABP5 had a high sensitivity (91.84%), high PPV (92.3%), and high NPV (85%) in the identification of LNM in CCa patients **(Table 2)**.

### FABP5 promotes CCa cell EMT, lymphangiogenesis, and LNM

**Figure 2:**
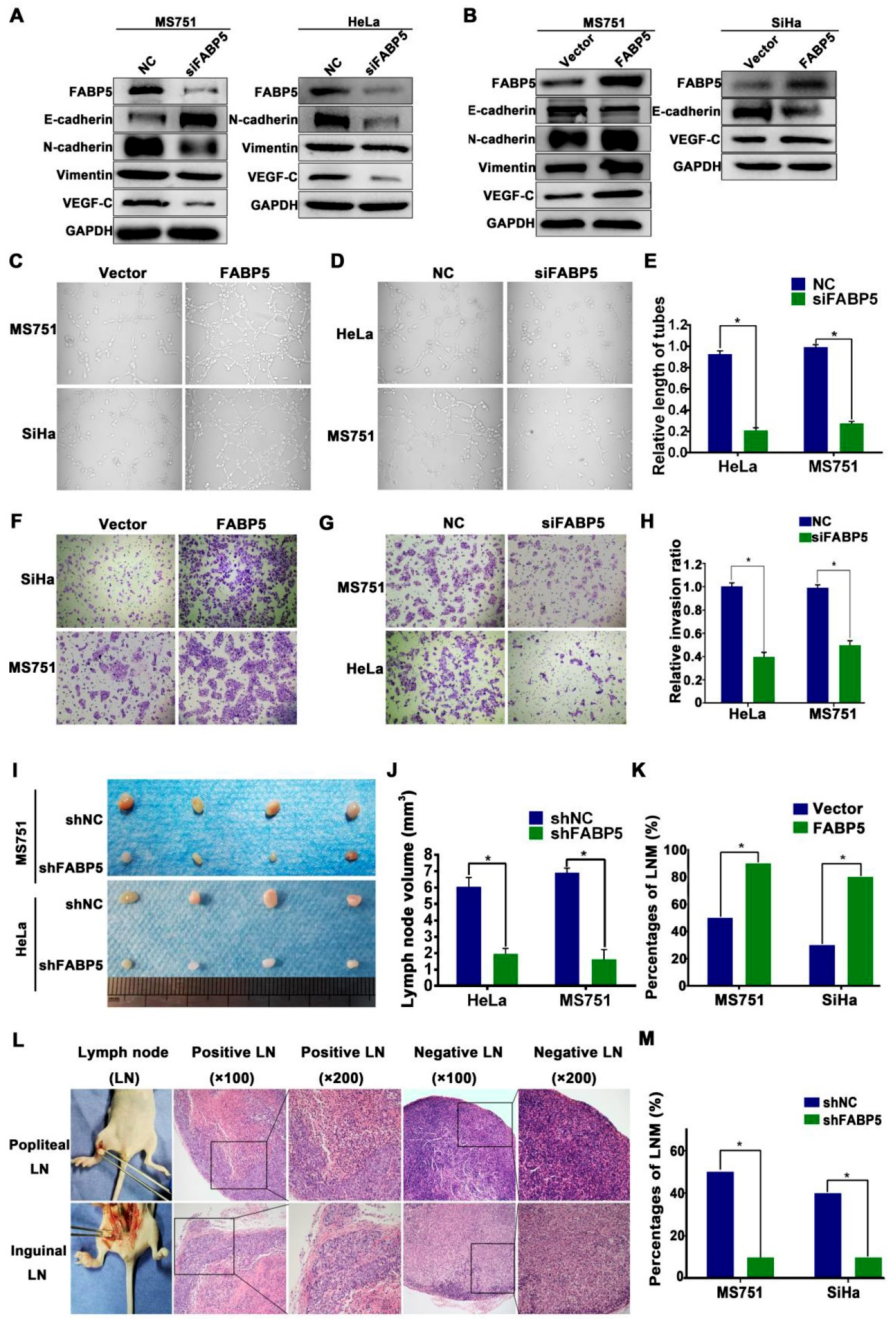
** FABP5 promotes CCa LNM via EMT and VEGF-C.** (A, B) Western blot analysis of EMT markers and VEGF-C in indicated cells with knockdown or overexpression of FABP5. (C-E) The effects of FABP5 on the tube formation of HLECs (×200). (F-H) Transwell assays were performed to investigate the effects of FABP5 on the invasion abilities of indicated cells (×200). (I, J) Representative images of popliteal lymph nodes and histogram analysis of the volume of lymph nodes (n = 10 mice per group). (K, M) The effects of FABP5 on the incidence of LNM in mouse model (n = 10 mice per group). (L) The representative pictures of HE staining of lymph nodes in different parts of mice. Error bars represent the mean ± S.D. of three independent experiments. *P < 0.05.

To investigate the potential promoting effects of FABP5 on LNM in CCa, we performed gain-of-function and loss-of-function experiments in CCa cells. As shown in Figure 1B, 1C, and 1E, the expression of FABP5 mRNA and protein was lowest in SiHa cells, moderate in HeLa cells, and highest in MS751 cells. The MS751 cell line, derived from a metastatic lymph node of cervical cancer patients, is associated with LNM in CCa. Therefore, we constructed an MS751 cell line with FABP5 overexpression and knockdown. The SiHa cell line was selected for overexpression of FABP5. The HeLa cell line was selected for FABP5 knockdown. The efficiencies of interference and overexpression were examined **(Figure S1A-B)**. We analysed the effects of FABP5 on the EMT process by Western blot analysis. The results showed significant upregulation of E-cadherin in MS751 cells and downregulation of vimentin and N-cadherin in MS751 and HeLa cells after FABP5 downregulation **(Figure 2A, Figure S1C-D)**. However, FABP5 overexpression led to a significant upregulation of vimentin and N-cadherin in MS751 cells and downregulation of E-cadherin in MS751 and SiHa cells **(Figure 2B, Figure S1E-F)**. Next, we evaluated the effect of FABP5 on the tube formation of HLECs, which is crucial for LNM in cancer. The culture supernatants of FABP5-knockdown cells significantly inhibited HLEC tube formation, while FABP5 overexpression promoted HLEC tube formation **(Figure 2C-E, Figure S1G)**. In addition, VEGF-C is a key factor involved in lymphangiogenesis. Our results showed that VEGF-C was increased at both the mRNA and protein levels in FABP5 overexpressing cells and decreased in FABP5 knockdown cells **(Figure 2A-B, Figure S1H-M)**.

To assess the potential role of FABP5 in regulating cell invasion, we performed a Transwell invasion assay. Our results indicated that FABP5 overexpression increased the invasion ability of MS751 and SiHa cells, whereas FABP5 knockdown significantly decreased the invasion ability of HeLa and MS751 cells **(Figure 2F-H, Figure S1N)**. To further examine the effects of FABP5 on LNM, a nude mouse LNM model was established. We found that the volume of popliteal lymph nodes was smaller in the FABP5-knockdown group and larger in the FABP5-overexpression group than in the control **(Figure 2I-J, Figure S1O)**. Moreover, FABP5 overexpression increased the incidence of LNM, whereas FABP5 knockdown significantly decreased the incidence of LNM **(Figure 2K-M)**.

**Figure 3:**
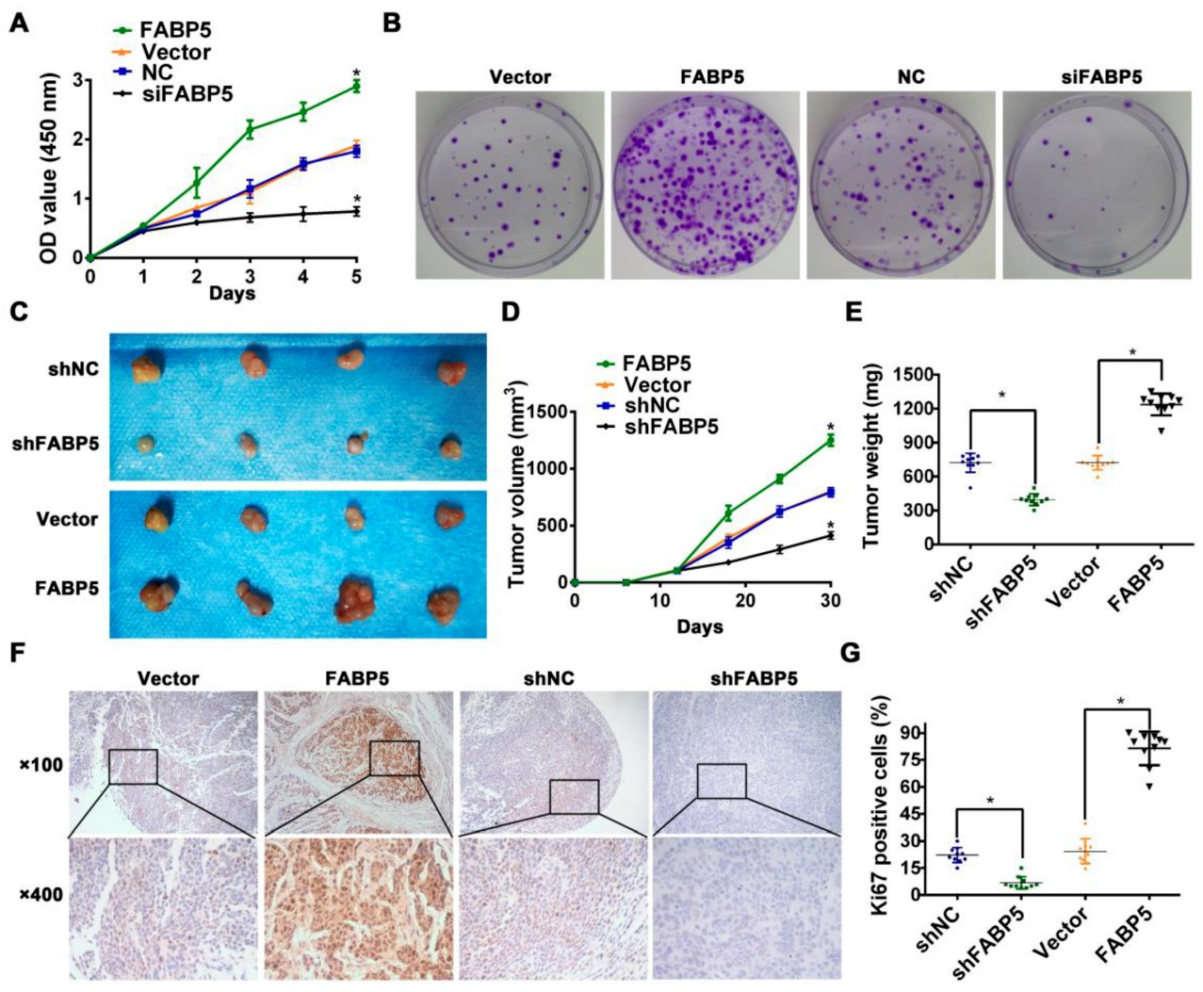
** FABP5 promotes CCa tumorigenesis.** (A) CCK8 assay was performed to investigate the effects of FABP5 on the proliferation abilities of CCa cells. (B) Colony formation assay was conducted to investigate the effects of FABP5 on the colony formation abilities of CCa cells (×100). (C-E) Representative images of gross appearance, measured volumes, and weights of subcutaneous tumors from nude mice inoculated with MS751 with FABP5 overexpression and knockdown (n = 10 mice per group). (F, G) Representative images and histogram analysis of IHC staining for Ki67 expression (n = 10 per group). Error bars represent the mean ± S.D. of three independent experiments. *P < 0.05.

Tumorigenicity is a major factor correlated with lymphangiogenesis and LNM in various solid tumours 32-34. Therefore, we investigated the tumorigenic effect of FABP5 in CCa. Colony formation assays and Cell Counting Kit-8 (CCK-8) proliferation assays revealed that FABP5 overexpression increased proliferation and colony formation in CCa cells. Conversely, FABP5 knockdown yielded the opposite effect on proliferation and colony formation **(Figure 3A-B)**.

Then, we constructed a subcutaneous xenograft model to evaluate the tumorigenic capacity of FABP5 in vivo. Our results indicated that FABP5 overexpression increased, while FABP5 knockdown decreased the tumour growth of CCa. Moreover, tumours in the FABP5 overexpression group were of greater weight and size than control groups. By contrast, tumors in the FABP5 knockdown group were of lower weight and size than those in the control group **(Figure 3C-E)**. Furthermore, the numbers of Ki67-stained cells were significantly decreased in the FABP5 knockdown group but increased in the FABP5 overexpression group **(Figure 3F-G)**. Collectively, these results show that FABP5 can promote lymphangiogenesis and LNM in CCa.

### FABP5 regulates fatty acid metabolism in CCa cells

**Figure 4:**
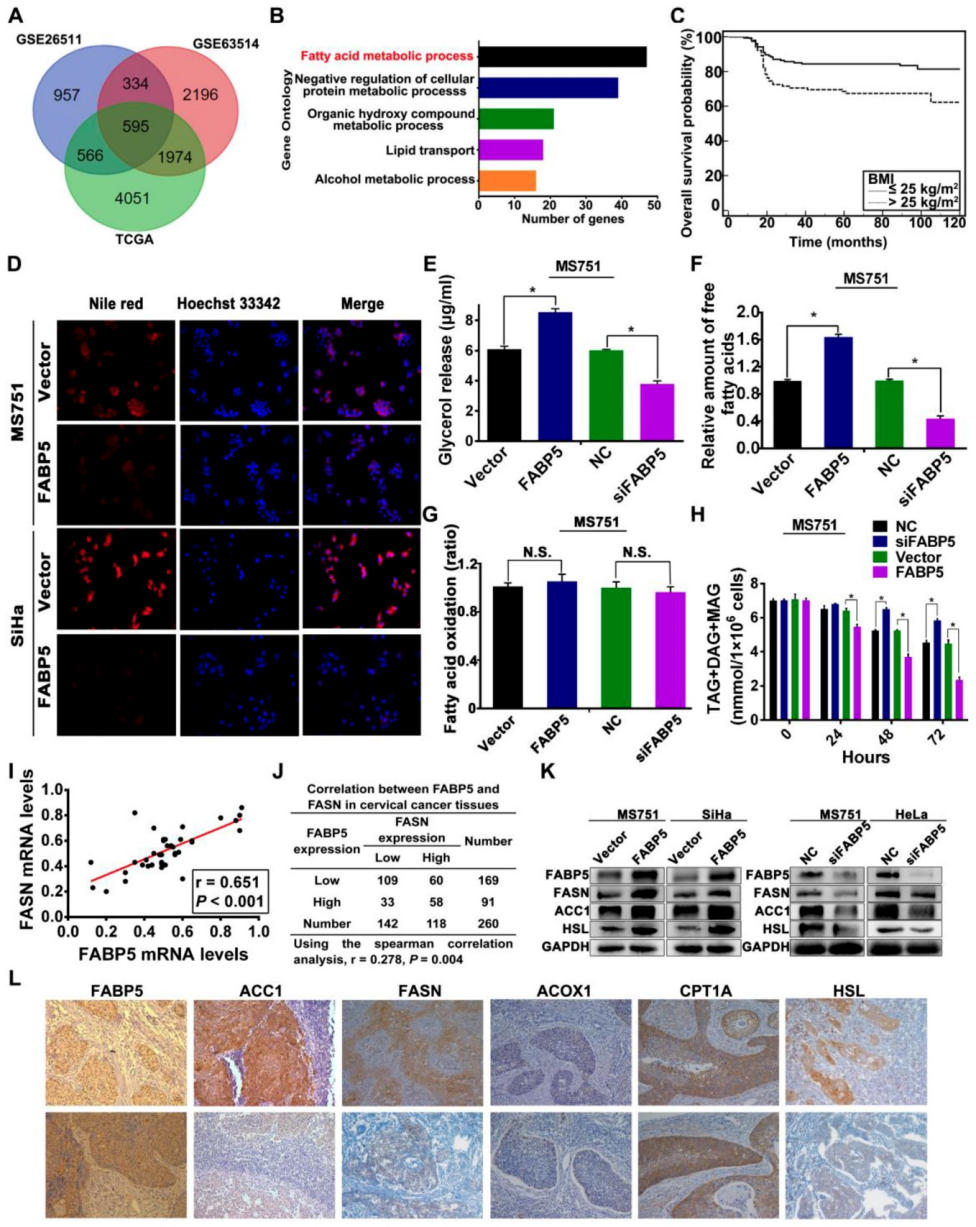
** Effects of FABP5 on FA metabolism in CCa.** (A) The genes correlated with FABP5 co-existing in TCGA, GSE26511, GSE63514 datasets. (B) The Gene Ontology analysis of genes correlated with FABP5 co-existing in TCGA, GSE26511, GSE63514 datasets. (C) Kaplan-Meier survival curves of OS in patients with CCa according to BMI. (D) LDs were detected using Nile red in indicated cells with FABP5 overexpression (×200). (E) Glycerol (F) free fatty acids (G) FAO (H) intracellular neutral lipids (TAG, DAG, and MAG) were measured in MS751 cells with FABP5 knockdown or overexpression. (I) Scatter plot analysis of correlation between mRNA expression of FABP5 and FASN in 40 CCa tissues. (J) The relationship between the protein expression of FABP5 and FASN was examined based on IHC analysis. (K) Western blot analysis for protein levels of FASN, ACC1, HSL, and FABP5 in the indicated cells. (L) Representative IHC images of the FA metabolic enzymes FASN, ACC1, HSL, ACOX1, CPT1A, and FABP5 in human CCa tissues (×200). Error bars represent the mean ± S.D. of three independent experiments. *P < 0.05.

To explore whether FABP5 is involved in reprogramming FA metabolism in CCa cells, we performed both bioinformatic analyses and experimental investigations. First, we analysed three public datasets of mRNA expression data from cervical cancer tissues, including RNA-seq data from the Cancer Genome Atlas (TCGA) and microarray data from the GSE26511 and GSE63514 datasets in the Gene Expression Omnibus (GEO). Spearman correlation analysis was used to determine the expression correlation between FABP5 and other genes. Gene Ontology (GO) and Kyoto Encyclopedia of Genes and Genomes (KEGG) enrichment analyses of genes correlated with FABP5 were performed. Gene sets with a false discovery rate (FDR) < 0.05 were considered statistically significant and ranked by the number of enriched genes. Gene Ontology analysis showed that genes that were significantly correlated with FABP5 mRNA expression were mainly involved in FA metabolism in all three datasets **(Figure 4A-B, Figure S2)**, suggesting that FABP5 might play a vital role in the reprogramming of FA metabolism in CCa. Furthermore, the multivariate regression analysis indicated that BMI > 25 kg/m^2^ was independently correlated with LNM in our cohort of CCa patients **(Table 1)**, and patients with BMI > 25 kg/m^2^ had significantly decreased RFS and OS **(Figure 4C, Figure S3R)**. Importantly, FABP5 protein expression was significantly associated with BMI (*P* = 0.013) **(Table S1)**. These findings suggest that FA metabolism is involved in the LNM of CCa and that FABP5 plays an important role in FA metabolism reprogramming of CCa.

To evaluate the effects of FABP5 on FA metabolism in CCa cells, we first examined intracellular lipid droplets (LDs) in CCa cells. LDs stained with Nile red were detected in the cytoplasm of MS751, SiHa, and HeLa cells. We next examined whether FABP5 affected the amounts of intracellular LDs in CCa cells. FABP5 knockdown induced the accumulation of LDs in MS751 and HeLa cell lines, whereas FABP5 overexpression led to the shrinkage of LDs in MS751 and SiHa cell lines **(Figure 4D, Figure S3A)**. LDs, as cytoplasmic organelles, store neutral lipids such as TAG, DAG, and MAG. Next, we assessed the levels of intracellular neutral lipids. Our data showed that CCa cells with FABP5 knockdown had significantly increased levels of intracellular neutral lipids, whereas those with FABP5 overexpression had decreased levels of intracellular neutral lipids **(Figure 4H, Figure S3P-Q)**. Furthermore, we examined the levels of intracellular FAs and glycerol to evaluate lipolytic activity. The levels of intracellular FAs and glycerol were significantly decreased in FABP5 knockdown cells but significantly increased in FABP5 overexpression cells **(Figure 4E-F, Figure S3B-E)**. These results indicate that FABP5 promotes lipolysis of LDs in CCa cells.

To further investigate the role of FABP5 in the deregulation of FA metabolism in CCa, we examined the expression of key FA metabolism enzymes involved in lipolysis, de novo fatty acid synthesis, and FAO, including hormone-sensitive lipase (HSL), fatty acid synthase (FASN), acetyl-CoA carboxylase 1 (ACC1), acyl-CoA oxidase 1 (ACOX1), and carnitine palmitoyltransferase-1A (CPT1A), in CCa tissues by IHC analysis **(Figure 4L)**. Spearman rank correlation analysis showed a significant positive correlation between the IHC scores of FABP5 and FASN (r = 0.278, *P* = 0.004) **(Figure 4J)**, FABP5 and ACC1 (r = 0.192, *P* = 0.002) **(Figure S3K)**, and FABP5 and HSL (r = 0.151, *P* = 0.015) **(Figure S3G)**. However, the expression of FABP5 protein had no significant correlation with ACOX1 or CPT1A **(Figure S3I, Figure S3M)**. Furthermore, we examined the mRNA levels of FABP5, HSL, FASN, ACC1, ACOX1 and CPT1A in 40 fresh CCa tissue samples. Scatter plot analysis indicated a positive correlation between FABP5 and FASN (r = 0.651, *P* < 0.001) **(Figure 4I)**, FABP5 and ACC1 (r = 0.370, *P* = 0.019) **(Figure S3J)**, and FABP5 and HSL (r = 0.609, *P* < 0.001) **(Figure S3F)** at the mRNA level, while there was no significant correlation between FABP5 and ACOX1 or CPT1A **(Figure S3H, S3L)**. To provide further validation, we analysed the expression of HSL, ACC1, and FASN in CCa cells at different FABP5 protein levels. Our results showed that significant upregulation of ACC1, FASN, and HSL protein levels was observed in FABP5 overexpressing CCa cells (SiHa and MS751), while FABP5 knockdown had the opposite effect on the expression of ACC1, FASN, and HSL protein in MS751 and HeLa cells **(Figure 4K, Figure S3V-Y)**. Furthermore, the rate of FAO, as measured by the conversion of radiolabelled palmitate to CO_2_, was not significantly changed with the alteration of FABP5 mRNA expression in CCa cells **(Figure 4G, Figure S3N-O)**. Because FAs can be taken up from the extracellular environment, we next analysed the activity of FA uptake. Our results showed that FA uptake was not markedly changed by FABP5 knockdown or overexpression **(Figure S3S-U)**, suggesting that FABP5 has little effect on FA uptake in CCa cells. Collectively, these results indicate that FABP5 promotes lipolysis and FA synthesis, resulting in an increase in intracellular FAs in CCa cells.

### FABP5 promotes LNM via FA metabolism reprogramming

**Figure 5:**
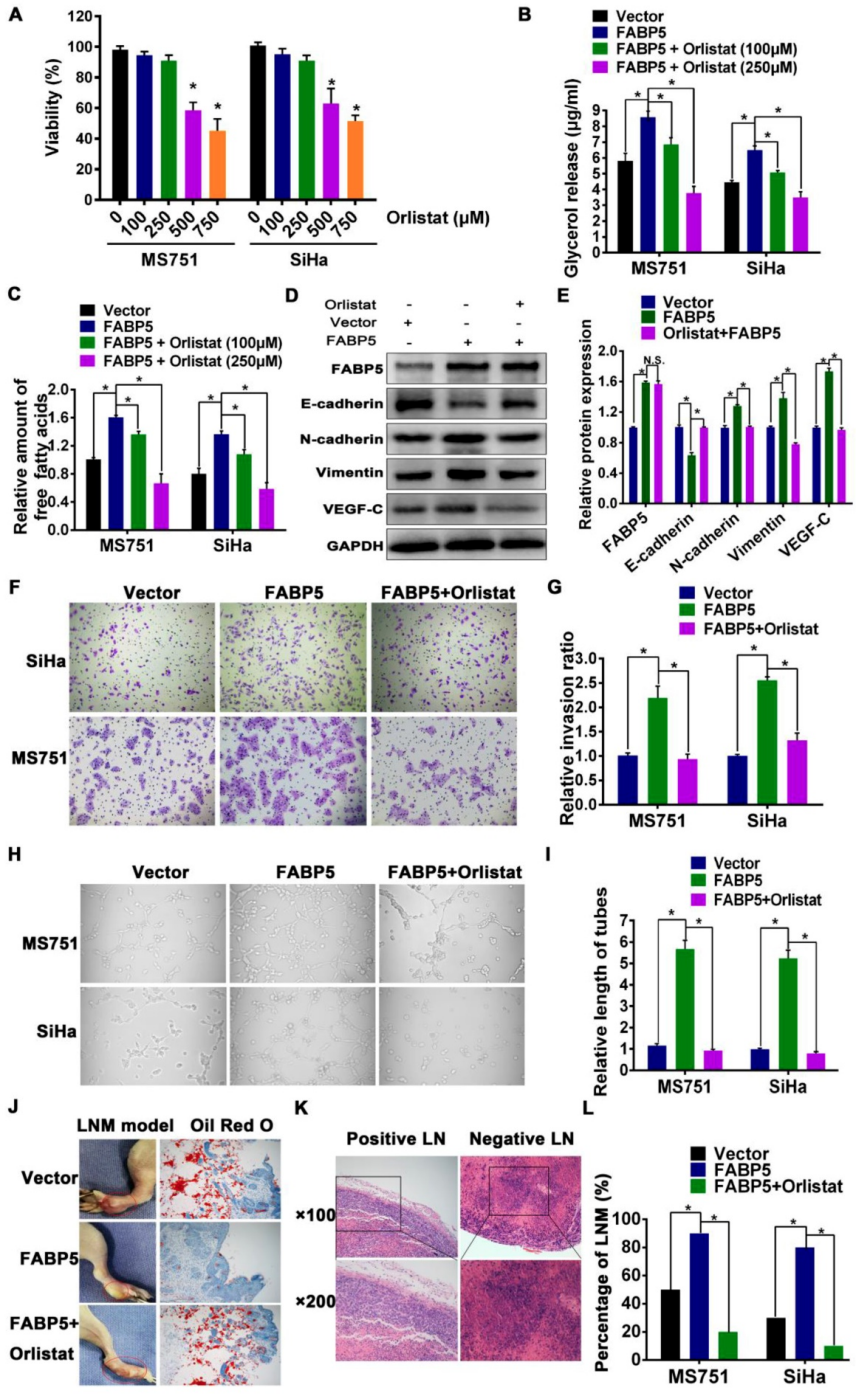
** Orlistat reversed the effect of FA metabolic alternation and inhibited FABP5-induced LNM in CCa.** (A) The effect of orlistat on proliferation of MS751 and SiHa cells by CCK-8 assays. MS751 and SiHa cells were treated by orlistat at the indicated concentrations for 48 hours. (B, C) 100 μM and 250 μM orlistat inhibited the amount of glycerol and FAs in indicated cells. (D) Western blot analysis of EMT markers and VEGF-C in indicated cells treated with orlistat (250 μM). (E) Quantification of western blot analysis of EMT markers and VEGF-C in indicated cells treated with orlistat (250 μM). (F, G) Transwell assays were performed to investigate the effects of orlistat (250 μM) on the invasion abilities of indicated cells (×200). (H, I) The effect of orlistat (250 μM) on the tube formation of HLECs (×200). (J) LDs were detected using Oil Red O staining in the foot pads of mice. (K) Representative pictures of HE staining of metastasis positive and negative lymph nodes. (L) The rate of LNM in mice with different treatment cells (n = 10 mice per group). Error bars represent the mean ± S.D. of three independent experiments. *P < 0.05.

Given that the important role of FA metabolism in cancer metastasis has been highlighted, we speculated that FABP5 promotes LNM in CCa by reprogramming FA metabolism. To test this hypothesis, we evaluated whether FA metabolism plays a pivotal role in FABP5-induced LNM. Orlistat, a potent and irreversible inhibitor of lipase and FASN 29, 35, was used to assess the role of FA metabolism in FABP5-induced LNM. First, to rule out the antiproliferative effects of orlistat, we investigated whether orlistat could modify the growth rate of MS751 and SiHa cells. Our data showed that the viability of MS751 and SiHa cells was not significantly affected by orlistat at 100 μM and 250 μM but was markedly reduced at 500 μM and 750 μM (Figure 5A). Therefore, we chose 100 μM and 250 μM as the administration doses of orlistat. Second, we explored the effect of orlistat treatment on FABP5 expression in CCa cells. Our data showed that FABP5 mRNA expression was not changed after the administration of orlistat in SiHa, MS751, and HeLa cells (Figure S5A-C). Subsequently, we found that orlistat reversed the effects of FABP5 on FA metabolism reprogramming in CCa cells. Our results showed that the content of intracellular glycerol, which represents lipolytic activity, was notably decreased in CCa cells after orlistat treatment **(Figure 5B)**. Moreover, the content of intracellular FAs was also significantly decreased after orlistat treatment in CCa cells **(Figure 5C)**. Furthermore, orlistat dramatically reversed the FABP5-induced invasion, EMT, and lymphangiogenesis of CCa cells **(Figure 5D-I)**. These results suggest that FABP5 promotes the invasion, EMT, and lymphangiogenesis of CCa cells by modulating FA metabolism.

In vivo, the FABP5 overexpression group exhibited a significantly increased incidence of LNM compared to that in the control group. Moreover, the specimens from the foot pads of mice in the FABP5 overexpression group contained fewer LDs than those in the control group and orlistat group **(Figure 5J)**. Importantly, orlistat reversed the increased trend of LNM in nude mouse LNM models with FABP5 overexpression **(Figure 5L)**. The presence of LNM was detected by HE staining **(Figure 5K)**.

### FABP5 promotes invasion, EMT, and lymphangiogenesis via excess intracellular FA-induced NF-κB signalling activation

**Figure 6:**
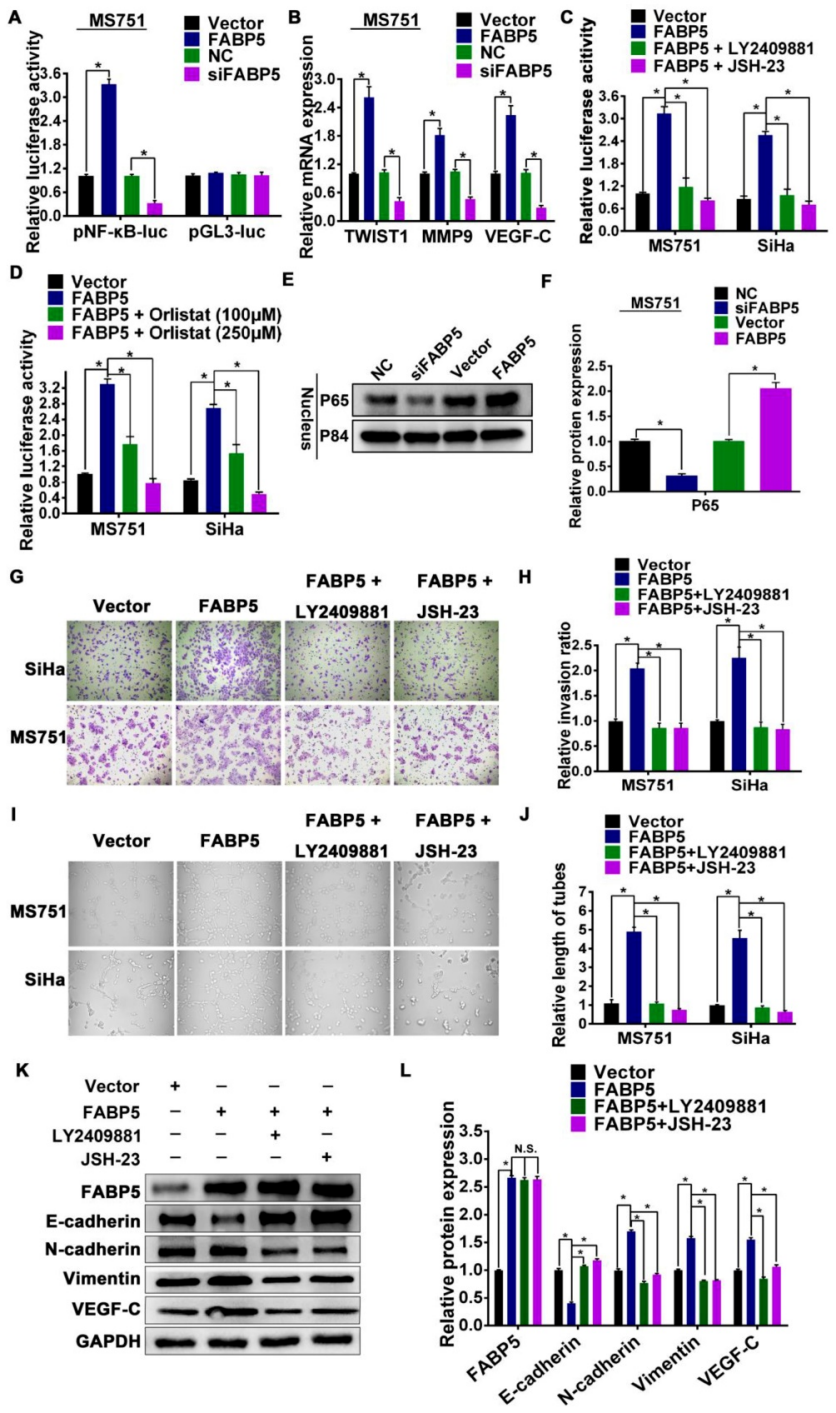
** FABP5 activates NF-κB signaling pathway.** (A) NF-κB transcriptional activity was assessed by luciferase reporter constructs in the indicated cells. (B) Real-time PCR analysis of TWIST1, MMP9 and VEGF-C in the indicated cells. (C) NF-κB signaling inhibitors LY2409881 (10 μM) and JSH-23 (10 μM) attenuated the stimulatory effect of FABP5 on NF-κB transcriptional activity in the indicated cells. (D) Orlistat attenuated the stimulatory effect of FABP5 on NF-κB transcriptional activity in the indicated cells. (E, F) Western blot of nuclear NF-κB/p65 expression in MS751 cells. The nuclear protein p84 was used as the nuclear protein markers. (G, H) NF-κB signaling inhibitors LY2409881 (10 μM) and JSH-23 (10 μM) attenuated the stimulatory effect of FABP5 on invasion ability in the indicated cells(×200) . (I, J) NF-κB signaling inhibitors LY2409881 (10 μM) and JSH-23 (10 μM) attenuated the stimulatory effect of FABP5 on the tube formation of HLECs (×200). (K, L) Western blot analysis of EMT markers and VEGF-C in indicated cells treated with LY2409881 (10 μM) and JSH-23 (10 μM). Error bars represent the mean ± S.D. of three independent experiments. *P < 0.05.

Our data showed that FABP5 increased the level of intracellular FAs via activation of lipolysis and FA synthesis in CCa cells. The level of intracellular FAs was increased in CCa cells overexpressing FABP5 **(Figure 4F, Figure S3D-E)**. Previous studies demonstrated that an increase in intracellular FAs can activate NF-κB signalling 17, 36-39. Therefore, we hypothesized that FABP5 can activate NF-κB signalling by increasing intracellular FAs in CCa. We found that FABP5 overexpression was significantly enhanced, while silencing FABP5 reduced NF-κB dependent luciferase activity in CCa cells **(Figure 6A, Figure S4A-B)**. Moreover, overexpression of FABP5 enhanced, while silencing of FABP5 reduced nuclear accumulation of NF-κB/p65 **(Figure 6E-F)**. Furthermore, overexpression of FABP5 increased the mRNA levels of multiple NF-κB signalling downstream metastasis-related target genes, including TWIST1, MMP9 and VEGF-C, in CCa cells. By contrast, silencing FABP5 repressed the mRNA expression of these downstream genes in CCa cells **(Figure 6B, Figure S4C-D)**. In addition, we further validated the effects of FABP5-mediated activation of NF-κB signalling using the FABP5 inhibitor SBFI-26 in CCa cells. We found that treatment with SBFI-26 significantly reduced NF-κB-dependent luciferase activity and the mRNA levels of TWIST1, VEGF-C, and MMP9 in CCa cells (Figure S5). Thus, these results demonstrate that FABP5 activates NF-κB signalling in CCa cells.

Next, we explored the functional significance of NF-κB signalling in the pro-metastatic role of FABP5 in CCa cells using the NF-κB signalling inhibitors LY2409881 and JSH-23. We found that LY2409881 and JSH-23 inhibited the NF-κB reporter activity in a dose-dependent manner in CCa cells **(Figure S4E-J)**. Moreover, our results indicated that the viability of MS751 and SiHa cells was not significantly affected by LY2409881 or JSH-23 at 5 μM and 10 μM but was markedly reduced at 15 μM, 20 μM, and 25 μM (Figure S4K-L). Therefore, we chose 10 μM as the administration dose of LY2409881 and JSH-23 in the subsequent experiments. We found that the stimulatory effect of FABP5 on NF-κB activity was attenuated by LY2409881 and JSH-23 **(Figure 6C)**. In addition, inhibition of NF-κB signalling by LY2409881 and JSH-23 impaired the promoting effect of FABP5 on invasion, EMT, and lymphangiogenesis **(Figure 6G-L)**. These results suggested that NF-κB signalling activation was essential for the pro-metastatic role of FABP5 in CCa cells. Furthermore, we further investigated the role of FAs in the FABP5-mediated activation of NF-κB signalling in CCa cells. As shown in **Figure 5C**, orlistat treatment inhibited the content of intracellular FAs in CCa cells. Importantly, after treatment with orlistat, FABP5-mediated activation of NF-κB signalling was attenuated in MS751 and SiHa cells **(Figure 6D)**. Collectively, these results indicate that, in CCa cells, FABP5 promotes invasion, EMT, and lymphangiogenesis via NF-κB signalling. Moreover, NF-κB signalling can be activated by the increase in intracellular FAs in CCa cells.

### FABP5 is a direct target of miR-144-3p

**Figure 7:**
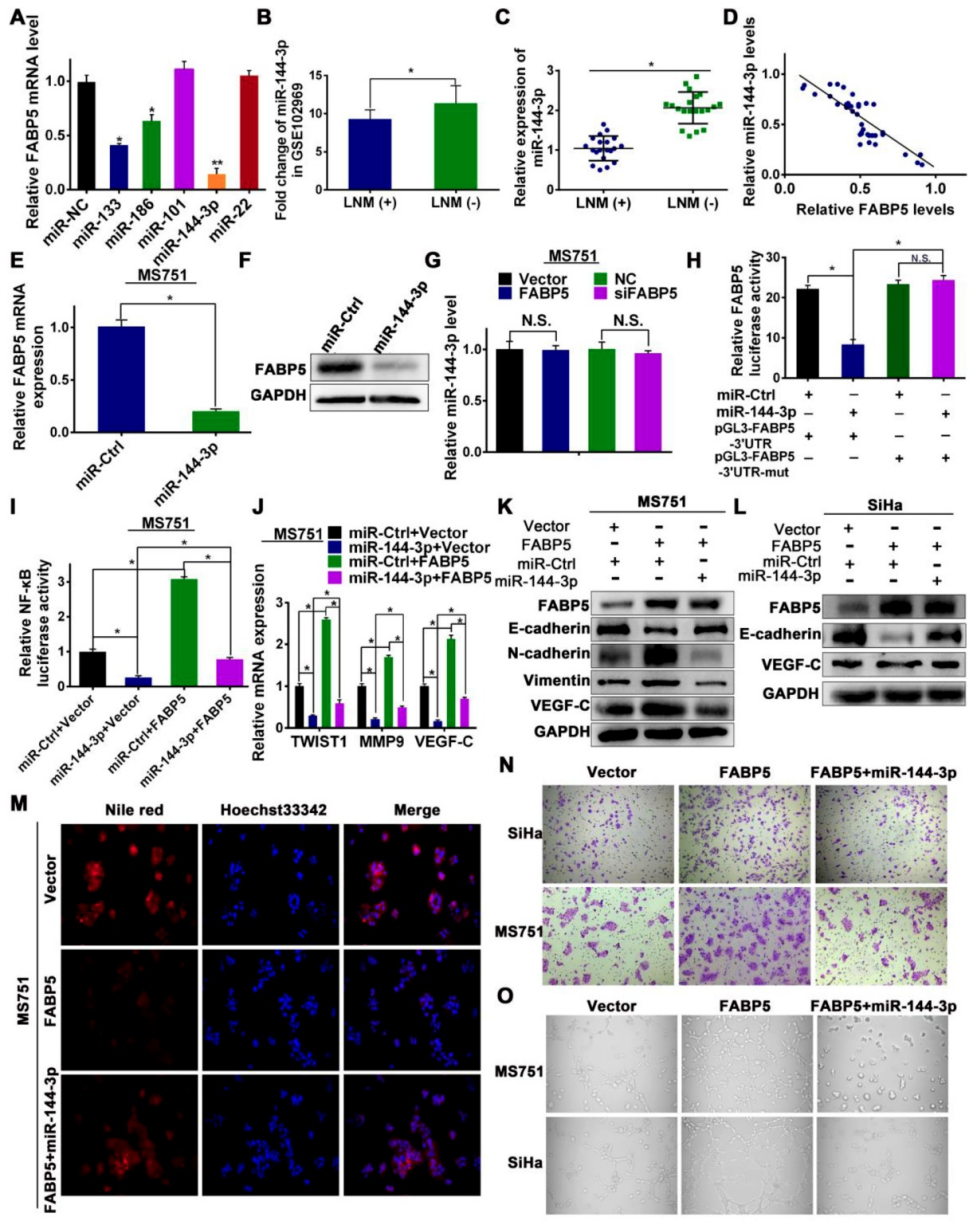
** Effect of miR-144-3p on FABP5 expression and tumor progression.** (A) Expression of FABP5 expression in MS751 cells transfected with miRNAs mimics, which were predicted by microRNA and TargetScan. (B, C) Expression of miR-144-3p in CCa tissues in the GSE102969 dataset and our tissue cohorts (40 fresh CCa tissues). (D) Scatter plot analysis of the correlation between mRNA expression levels of FABP5 and miR-144-3p in our tissue cohorts. (E) Effect of miR-144-3p mimics transfection specifically on FABP5 expression level (RNA) in MS751 cells. (F) Effect of miR-144-3p mimics transfection on FABP5 expression level (protein) in MS751 cells. (G) Effect of FABP5 on miR-144-3p expression level in MS751 cells. (H) FABP5 transcriptional activity of MS751 cells cotransfected with pGL3-FABP5-3'UTR or pGL3-FABP5-3'UTR-mut reporter plasmids and miR-144-3p mimics. (I) NF-κB transcriptional activity was assessed by luciferase reporter constructs in the indicated cells. (J) Expression of NF-κB signaling downstream target genes (TWIST1, MMP9 and VEGF-C) in the indicated cells. (K, L) Western blot analysis of EMT markers and VEGF-C in indicated cells treated with miR-144-3p mimics. (M) Lipid droplets were detected using Nile red in MS751 cells treated with miR-144-3p mimics (×200). (N) miR-144-3p mimics attenuated the stimulatory effect of FABP5 on invasion ability in the indicated cells (×200). (O) miR-144-3p mimics attenuated the stimulatory effect of FABP5 on the tube formation of HLECs (×200). Error bars represent the mean ± S.D. of three independent experiments. *P < 0.05.

Next, we explored the upstream regulatory machinery of FABP5. Two software systems (microRNA and TargetScan) were used to predict potential complementary base pairing between FABP5 and miRNAs. Five miRNAs, including miR-133, miR-186, miR-101, miR-144-3p, and miR-22, were co-existed in the data from the two software. We transfected MS751 cells with mimics of all predicted miRNAs, and miR-144-3p mimics had the most significant effect on FABP5 mRNA expression **(Figure 7A)**. Therefore, we chose miR-144-3p for further study. First, we probed a Gene Expression Omnibus dataset (GSE102969) and investigated the expression of miR-144-3p in CCa tissues. GSE102969 dataset analysis revealed that miR-144-3p was expressed significantly lower in CCa tumours with LNM than in those without LNM **(Figure 7B)**. Furthermore, in our 40 independent fresh CCa tissues (20 with LNM and 20 without LNM), the expression of miR-144-3p was significantly lower in CCa tumours with LNM than in those without LNM **(Figure 7C)**. Importantly, a significant inverse correlation between FABP5 and miR-144-3p was found in CCa tumours **(Figure 7D)**. Next, we transfected MS751 cells with miR-144-3p mimics and found significantly decreased expression of FABP5 at the mRNA and protein levels **(Figure 7E-F, Figure S6C)**. A similar effect was observed in SiHa and HeLa cells **(Figure S6A-B)**. However, the miR-144-3p level was not significantly different in CCa after FABP5 overexpression or knockdown** (Figure 7G, Figure S6D-E)**. These results suggest that FABP5 might be the downstream effector of miR-144-3p. In addition, because FABPs are a family of highly homologous proteins, we further explored the relationship between miR-144-3p and FABP3, FABP4, and FABP7. We first investigated TargetScan and found no potential complementary base pairing between miR-144-3p and FABP3, FABP4, and FABP7. Next, we transfected MS751, SiHa, and HeLa cells with miR-144-3p mimics and found no significantly decreased expression of FABP3, FABP4, and FABP7 at the mRNA level (Figure S7).

To confirm whether miR-144-3p binds to the 3'-UTR of FABP5 mRNA, a dual-luciferase reporter assay was performed. The relative luciferase activity was significantly reduced in CCa cells cotransfected with pGL3-FABP5-3'UTR and miR-144-3p mimic **(Figure 7H)**. When 3'-UTR of FABP5 mRNA was mutated, these repressive effects were abrogated **(Figure 7H)**, suggesting that miR-144-3p binds to the 3'-UTR of FABP5 mRNA.

To explore whether the functions of FABP5 were regulated by miR-144-3p, we performed a rescue experiment. MiR-144-3p mimics efficiently attenuated the FABP5-induced promotion of lipolysis and fatty acid synthesis in CCa cells, as revealed by the level of glycerol **(Figure S6G-H)**, Nile red staining **(Figure 7M, Figure S6L)**, and intracellular free FAs **(Figure S6I)**. Increased NF-κB activity and VEGF-C, TWIST1, and MMP9 mRNA expression induced by FABP5 overexpression were rescued by miR-144-3p mimics treatment **(Figure 7I-J, Figure S6F, 6J)**. Moreover, the miR-144-3p mimics reversed the FABP5-stimulated mesenchymal cell phenotype **(Figure 7K-L, Figure S6M-N)**. Furthermore, we validated that miR-144-3p mimics could abrogate the effects of FABP5 in promoting cell invasion and HLEC tube formation **(Figure 7N-O, Figure S6K, S6O)**. These observations showed that the effects of FABP5 on FA metabolism, EMT, lymphangiogenesis, and invasion were abrogated by miR-144-3p, in accordance with the suppression of FABP5 mRNA and protein expression by miR-144-3p.

### Hypoxia regulates the expression of miR-144-3p and FABP5

**Figure 8:**
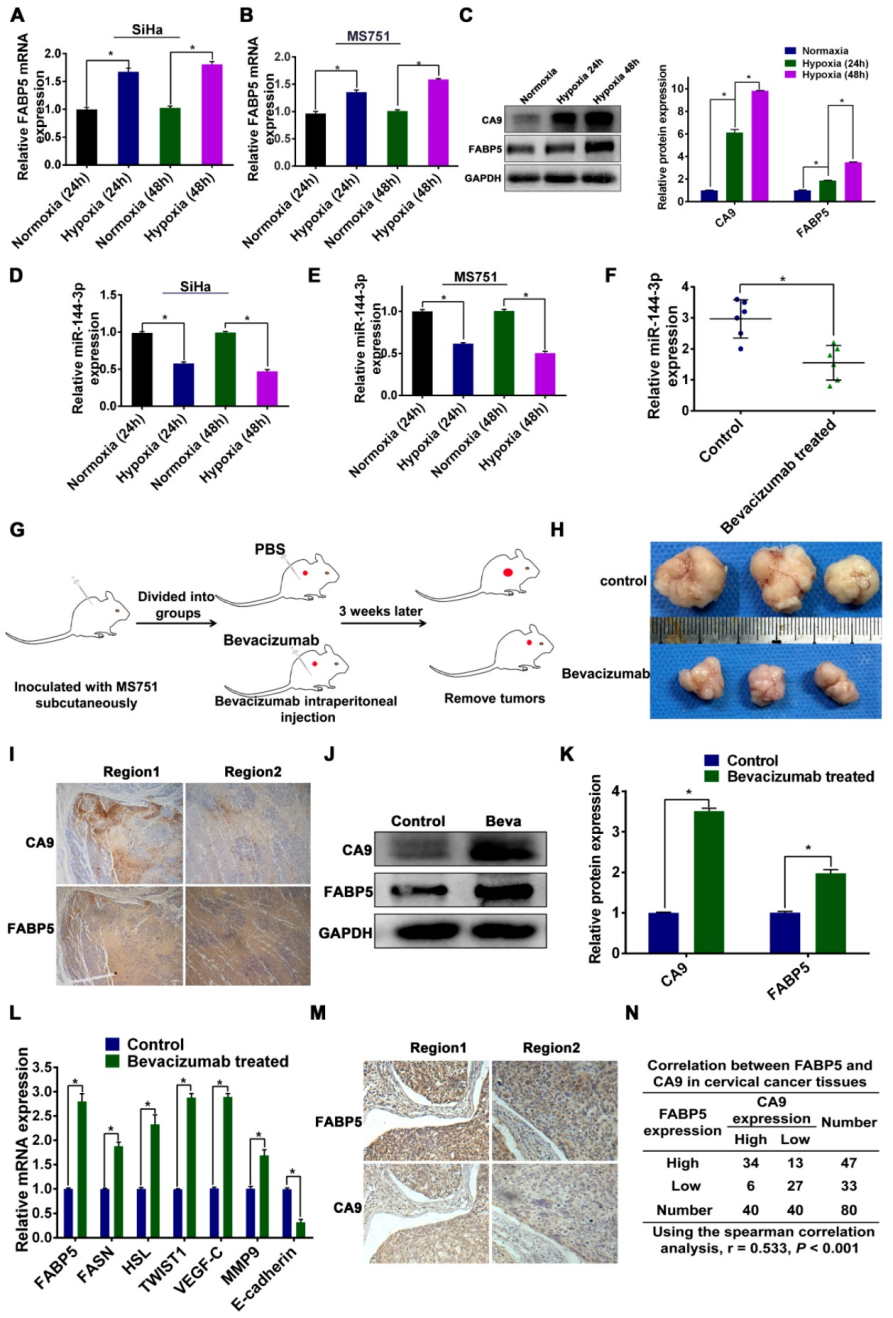
** Hypoxia in the regulation of miR-144-3p and FABP5 expression.** (A-C) Effect of hypoxia on FABP5 mRNA and protein expression in the indicated cells at 24 h and 48 h. (D, E) Effect of hypoxia on miR-144-3p expression in the indicated cells at 24 h and 48 h. (F) miR-144-3p expression levels in tumour tissues from mice treated with bevacizumab, which is known to induce hypoxia (n = 6 mice per group). (G) Schematic representation of the establishment of the xenograft model. (H) Representative images of gross appearance of subcutaneous tumours from nude mice treated with PBS and bevacizumab. (I) Co-localization of FABP5 and CA9 (a hypoxia marker) in tumour tissues taken from mice treated with bevacizumab. Representative images are shown. (J) Western blot analysis of FABP5 and CA9 protein in tumour tissues from mice treated with bevacizumab and control. (K) Quantification of western blot analysis of FABP5 and CA9 protein in tumours tissues from mice treated with bevacizumab and control. (L) Relative mRNA expression of FABP5, FASN, HSL, TWIST1, VEGF-C, MMP9, and E-cadherin in tumour from mice treated with bevacizumab and PBS. (M) Co-localization of FABP5 and CA9 in tumour tissues taken from CCa patients. Representative images are shown. (N) The spearman correlation analysis of CA9 and FABP5 expression in CCa tumours. Error bars represent the mean ± S.D. of three independent experiments. *P < 0.05.

Crosstalk between tumour cells and the microenvironment affects cellular lipid metabolism and tumour progression. Hypoxia, as a hallmark microenvironment of cancer, has a major role in FA metabolic reprogramming and the metastasis of tumour cells 40. Our results showed that FABP5 can reprogram FA metabolism and promote LNM. Moreover, a previous study showed that hypoxia upregulates the expression of FABP5 mRNA and protein in human aortic endothelial cells 41. In addition, hypoxia induces significant downregulation of miR-144-3p in prostate cancer cells 42. Therefore, we postulated that hypoxia might modulate the expression of miR-144-3p and FABP5 in CCa cells. To examine the effect of hypoxia on the expression of FABP5 and miR-144-3p, we cultured MS751 and SiHa cells under normoxic and hypoxic conditions. Our results indicated that the expression of FABP5 mRNA and protein was significantly increased in hypoxic conditions **(Figure 8A-C)**. Furthermore, the expression of miR-144-3p was markedly lower in hypoxic conditions than in normoxic conditions **(Figure 8D-E)**.

To validate our in vitro results, we examined the relationship between FABP5 expression and hypoxia in an animal xenograft model (**Figure 8G-H**). Bevacizumab, which targets vascular endothelial growth factor, has been shown to induce hypoxia in tumours 15, 43. Hence, we compared miR-144-3p expression between bevacizumab-treated and control tumours. The expression of miR-144-3p was significantly lower in tumours from mice treated with bevacizumab than in mice treated with the control **(Figure 8F)**. The mRNA and protein expression of FABP5 was significantly higher in tumours from mice treated with bevacizumab than in mice treated with the control **(Figure 8J-L)**.Moreover, we examined the expression of CA9 (a hypoxia marker) and FABP5 in tumour sections taken from xenograft mouse tumours by IHC. Our results showed that areas of FABP5 staining overlapped with areas of CA9 staining, suggesting that hypoxic areas in these tumours strongly expressed FABP5 **(Figure 8I)**.

To further validate these results in a clinical setting, we performed IHC analysis in clinical samples from 40 CCa patients (20 with LNM and 20 without LNM). We first stained the samples with anti-CA9 and randomly chose high and low expression fields for each specimen. The same fields in another slide were stained with an antibody against FABP5 **(Figure 8M)**. We then inspected the staining intensity of FABP5 in these CA9-high and CA9-low expression regions. As shown in Figure 8N, 34 of 40 CA-9 high expression regions had high FABP5 expression, and 6 regions had low FABP5 expression. On the other hand, 27 of 40 CA-9 low expression regions were also FABP5 low expression (*P* < 0.001). These results indicated that FABP5 was preferentially expressed in the region of hypoxia in CCa, which was consistent with our in vitro data. We further examined the expression of FABP5 and downstream molecules in subcutaneous tumours. We found that the mRNA expression levels of FABP5, FASN, HSL, TWIST1, VEGF-C, and MMP9 were significantly higher in tumours of the bevacizumab group than those of the control group. The mRNA expression level of E-cadherin was lower in tumours of the bevacizumab group than in those of the control group (**Figure 8L**). Taken together, our results indicate that the expression of FABP5 and miR-144-3p is regulated by hypoxia in CCa.

## Discussion

Lymph node metastasis (LNM) confers a poor prognosis in CCa patients, as effective treatment modalities are currently lacking for these patients 44. Therefore, elucidation of the molecular mechanisms underlying LNM may provide clinical preventive and therapeutic strategies for CCa patients with LNM. However, the precise mechanism of LNM in CCa is largely unknown. Herein, we investigated the critical role of cross-talk between FABP5 and FA metabolism in LNM of CCa. We demonstrated that FABP5 was upregulated in CCa with LNM, correlated with poor prognosis and was an independent predictor of LNM in CCa patients. Moreover, FABP5 promoted lipolysis of LDs and fatty acid synthesis in CCa cells. Subsequently, the increase in intracellular FAs produced by FA metabolic alterations activated the NF-κB pathway, resulting in LNM of CCa.

**Figure 9:**
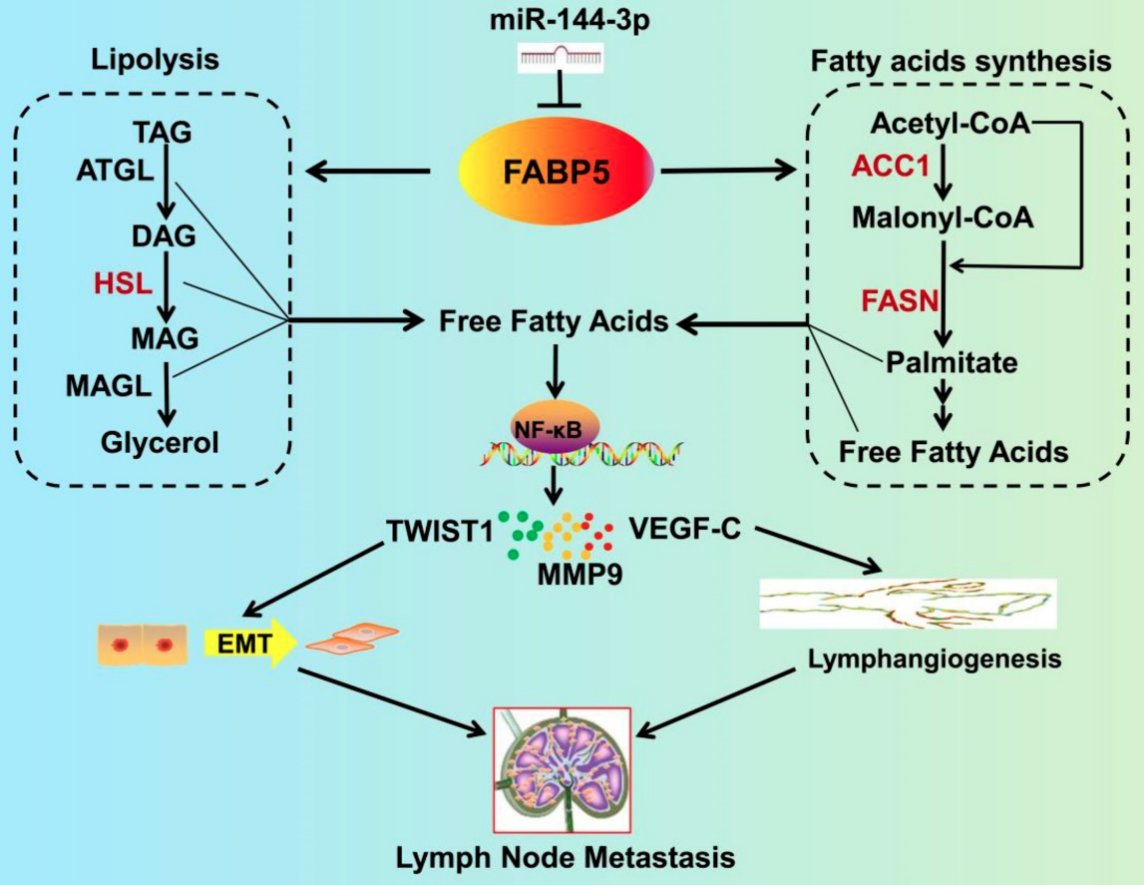
** Schematic illustration showing the proposed mechanism by which FABP5 promotes LNM of CCa by regulating FA metabolism.** FABP5 is directly targeted by miR-144-3p. Then, FABP5 promotes LD lipolysis and FA synthesis in CCa cells. Subsequently, the increase in intracellular FAs produced by FA metabolic alteration activates the NF-κB pathway, resulting in EMT, lymphangiogenesis, and LNM in CCa.

Furthermore, we investigated the upstream regulation of FABP5 and found that FABP5 is a direct target of miR-144-3p, which is downregulated by hypoxia **(Figure 9)**. Importantly, inhibition of FABP5 dramatically repressed LNM in vivo and prolonged the survival times of tumour-bearing mice. These findings provide mechanistic and translational insights into the FA metabolism-dependent pathway by which FABP5 promotes LNM and support the emergence of FABP5 as a novel diagnostic and therapeutic target in CCa.

Several independent studies have shown that FABP5 is involved in the development and progression of various cancers 16-24. However, the biological role of FABP5 in LNM remains unclear. Herein, in agreement with our previous study, we found that FABP5 was upregulated in the primary tumours of CCa with LNM and that its expression significantly was correlated with LNM. Importantly, we found that FABP5 was an independent predictor of LNM in multivariate logistic analysis. Moreover, the combination of FABP5, BMI, and LVSI could efficiently discriminate between CCa patients with LNM and CCa patients without LNM (AUC = 0.913) with high sensitivity (91.84%), high PPV (92.3%), high NPV (85%), and moderate specificity (71.56%). As primary CCa tumour samples can be easily obtained by transvaginal biopsy so that the expression levels of FABP5 can be tested before treatment, the combination of FABP5, BMI, and LVSI can be applied to assess LNM in CCa patients before the first treatment. These results suggest that clinical examination of FABP5 before treatment can be utilized for the detection of LNM and may be helpful in the clinical decision-making process. Consistent with our previous study, we found that FABP5 overexpression promoted CCa cell proliferation and invasion in vitro and in vivo 14. Furthermore, our data indicated that FABP5 overexpression promoted CCa cell EMT, lymphangiogenesis, and LNM in vitro and in vivo. Strikingly, knockdown of FABP5 dramatically inhibited tumour-associated LNM in vivo, suggesting that FABP5 may represent a potential molecular target for clinical intervention in CCa patients.

The alterations in intracellular metabolic intermediates that can accompany cancer-associated metabolic reprogramming have profound effects on tumour progression. Accumulating evidence has demonstrated that FAs are required as energy sources and cellular signalling molecules that are crucial for cancer metastasis 45, 46. In addition, fatty acid-binding proteins (FABPs) are involved in FA metabolism during cancer metastasis 16, 47. Thus, we wondered whether FABP5 could modulate FA metabolism to promote LNM in CCa. Herein, we found that FABP5 increased the level of intracellular FAs via the activation of LDs lipolysis and FA synthesis in CCa cells. Importantly, inhibition of FA metabolism alteration showed promising suppressive effects on LNM in FABP5-overexpressing CCa cells, indicating the critical role of FA metabolism reprogramming in FABP5-induced LNM of CCa. Therefore, inhibiting the function of FABP5 by targeting FA metabolism reprogramming might be a new strategy for treating CCa patients with LNM.

Another important finding in the present study was that FABP5 activated the NF-κB pathway by increasing of intracellular FAs in CCa. Aberrant activation of the NF-κB pathway is associated with the progression of cancer 48-52. Key cellular processes governed by the effectors of the NF-κB pathway are crucial in the metastasis of cancer 53. Although the NF-κB pathway plays an important role in the LNM of gallbladder cancer and breast cancer 54-56, NF-κB pathway activation in the LNM of CCa remains unknown. Herein, we found that FABP5 activated the NF-κB pathway by excess intracellular FAs in CCa cells. Consistent with our study, Senga and colleagues indicated that excess intracellular FAs induced the activation of NF-κB pathway in prostate and breast cancer cells 17. In addition, we found that FABP5 increased the expression of NF-κB pathway downstream metastasis-related target genes, such as TWIST1, MMP9 and VEGF-C. This result suggests that FABP5 induces the expression of NF-κB target genes to promote cancer cell aggressiveness and contribute to LNM in CCa. Moreover, inhibition of NF-κB signalling attenuated the LNM-promoting effect of FABP5 in CCa. These findings indicate that FABP5 constitutively activates the NF-κB pathway, which is crucial for LNM of CCa and expands the current knowledge on NF-κB pathway regulation in CCa.

Hypoxia is a common feature in solid tumours and has been linked to cancer aggressiveness 57-60. Herein, we found that hypoxia downregulated the expression of miR-144-3p, which subsequently increased the expression of FABP5 in CCa. In line with our findings, Gu et al. found that hypoxia induced miR-144-3p downregulation in prostate cancer 42. Moreover, recent literature showed that the expression of miR-144-3p was significantly lower in CCa tissues with LNM than in those without LNM 61. We found that miR-144-3p inhibited tumour cell invasion, EMT, and lymphangiogenesis, suggesting that miR-144-3p may play an important role in the LNM of CCa. In addition, our findings provide a new understanding of the upstream regulatory machinery of FABP5. However, miR-144-3p is not the only upstream regulator of FABP5 because at least three miRNAs were predicted to form complementary base pairings with FABP5. Thus, further investigations are needed to explore the regulatory networks of FABP5 in the LNM of CCa.

In summary, our findings demonstrate that FABP5, as a promising predictor of LNM, promotes LNM by reprogramming FA metabolism in CCa. Moreover, the expression and biological function of FABP5 can be regulated by miR-144-3p in hypoxia. Thus, our study not only identifies a crucial mechanism of cross-talk between FABP5 and FA metabolism that promotes LNM but also supplies a potential early diagnostic biomarker and therapeutic target for CCa patients with LNM.

## Figures and Tables

**Table 1:** Multivariate logistic regression analyses of factors associated with LNM.

Variables	HR (95% CI)	*P*
LVSI		< 0.001
Negative (reference)	1	
Positive	41.589 (12.936-133.706)	
BMI (kg/m^2^)		0.001
≤ 25	1	
> 25	4.402 (1.786-10.847)	
FABP5		< 0.001
Low (reference)	1	
High	9.097 (3.440-24.054)	

HR, hazard ratio; 95% CI, 95% confidence interval; FIGO, the International Federation of Gynaecology and Obstetrics; LVSI, lymphovascular space invasion. For the stepwise multivariate analysis, Forward LR method was used to select significant variables. Variables entered for analysis were: age, BMI, tumour size, differentiation, FIGO stage, LVSI, stomal invasion, parametrial invasion, vaginal invasion, FABP5.

**Table 2:** Sensitivity, specificity, AUC, positive and negative predictive values of factors associated with LNM.

Factors	Sensitivity (95% CI) (%)	Specificity (95% CI) (%)	PPV (95% CI) (%)	NPV (95% CI) (%)	AUC (95% CI)
LVSI	55.10 (40.2-69.3)	97.16 (93.9-98.9)	81.8 (64.2-93.2)	90.3 (85.7-93.8)	0.761 (0.705-0.812)
BMI	73.47 (58.9-85.1)	68.72 (62.0-74.9)	35.3 (26.1-45.4)	91.8 (86.3-95.6)	0.711 (0.652-0.765)
FABP5	71.43 (56.7-83.4)	73.46 (67.0-79.3)	38.5 (28.4-49.3)	91.7 (86.5-95.4)	0.724 (0.666-0.778)
FABP5+LVSI+BMI	91.84 (80.4-97.7)	71.56 (93.9-98.9)	92.3 (64-99.8)	85 (79.9-89.2)	0.913 (0.872-0.944)

PPV, positive predictive value; NPV, negative predictive value; AUC, the area under the receiver operating characteristic curve; 95% CI, 95% confidence interval; BMI, body mass index; LVSI, lymphovascular space invasion.

## Abbreviations

CCa: cervical cancer
FABP5: fatty acid-binding protein 5
LNM: lymph node metastasis
miR-144-3p: microRNA-144-3p
EMT: epithelial-mesenchymal transition
qRT-PCR: quantitative real time polymerase chain reactions
FA: fatty acid
FAs: fatty acids
HLECs: Human lymphatic endothelial cells
NCC: normal cervical epithelial cells
VEGF-C: vascular endothelial growth factor C
ATCC: the American Type Culture Collection
ELISA: enzyme-linked immunosorbent assay
FBS: foetal bovine serum
TAG: intracellular triacylglycerol
DAG: diacylglycerol
MAG: monoacylglycerol
HE: hematoxylin and eosin
RFS: recurrence-free survival
OS: overall survival
LVSI: lymphovascular space invasion
ROC: receiver operating characteristic
AUC: the area under the ROC curve
PPV: positive predictive value
NPV: negative predictive value
BMI: body mass index
FIGO: the International Federation of Gynecology and Obstetrics
CCK-8: cell counting kit-8
LDs: lipid droplets
FAO: fatty acid oxidation
HSL: hormone-sensitive lipase
FASN: fatty acid synthase
ACC1: acetyl-CoA carboxylase 1
ACOX1: acyl-CoA oxidase 1
CPT1A: carnitine palmitoyltransferase-1A
TCGA: The Cancer Genome Atlas
GEO: the Gene Expression Omnibus
FDR: false discovery rate
3'-UTR: 3'-untranslated region
IHC: immunohistochemistry
ceRNA: competing endogenous RNA
