# Supplementary material for: FABP5 promotes lymph node metastasis in cervical cancer by reprogramming fatty acid metabolism
Source: Theranostics. 2020 May 17;10(15):6561–80. doi: 10.7150/thno.44868 (PMC7295046; doi:10.7150/thno.44868)
Supplement: Supplementary file 1 — Supplementary figures and tables. [file thnov10p6561s1.pdf]

Table S1. Clinicopathological data of cervical cancer patients included in the IHC study.

No.	Age (years)	LNM	Tumour size (cm)	FIGO stage	Differentiation	BMI (kg/m^2)	LVSI	Parametrium invasion	Vaginal invasion	Stromal invasion
1	46	negative	3.5	IB2	G1	25.6	Negative	negative	positive	less than 1/2
2	41	positive	5	IA2	G1	27	Negative	negative	negative	more than 1/2
3	39	positive	4.5	IIA1	G2	26.9	Negative	negative	negative	less than 1/2
4	30	negative	4.5	IB1	G3	24.1	Negative	negative	negative	less than 1/2
5	37	negative	4.5	IIA2	G3	29.1	Negative	negative	negative	less than 1/2
6	60	negative	2	IB1	G3	23.5	Negative	negative	negative	less than 1/2
7	50	negative	1.5	IIA2	G1	21.5	Negative	negative	positive	less than 1/2
8	45	negative	5	IA2	G3	22.7	positive	positive	negative	more than 1/2
9	36	negative	2	IB2	G3	23.1	Negative	negative	negative	more than 1/2
10	31	negative	5	IIA2	G3	23.8	Negative	negative	negative	less than 1/2
11	48	positive	5.5	IB2	G1	30	positive	negative	negative	more than 1/2
12	35	positive	5	IIA1	G3	28	Negative	positive	negative	less than 1/2
13	49	positive	5.5	IIA1	G1	22.5	Negative	negative	negative	more than 1/2
14	43	negative	3.5	IB1	G3	21.8	Negative	negative	positive	less than 1/2
15	34	negative	1.5	IIA1	G1	29.1	Negative	negative	negative	more than 1/2
16	28	negative	3	IB1	G1	21.9	Negative	positive	negative	less than 1/2
17	64	negative	3	IB2	G3	24.5	Negative	negative	negative	more than 1/2
18	29	negative	0.5	IB1	G1	32	Negative	negative	positive	less than 1/2
19	40	negative	0.5	IB1	G3	31.5	Negative	negative	negative	less than 1/2
20	34	negative	1.5	IB1	G3	24.1	Negative	negative	negative	more than 1/2
21	47	positive	4.5	IB2	G2	32	Negative	negative	negative	less than 1/2
22	27	negative	2	IIA1	G3	23.5	Negative	negative	positive	less than 1/2
23	34	negative	5	IIA1	G2	23.1	Negative	negative	negative	more than 1/2
24	46	negative	3	IA2	G3	22.8	Negative	negative	negative	more than 1/2
25	42	negative	2	IB1	G1	22.9	Negative	negative	negative	less than 1/2
26	35	positive	5	IIA1	G2	28.4	Negative	negative	negative	more than 1/2
27	38	negative	5	IB2	G3	23.5	Negative	negative	negative	less than 1/2
28	34	negative	2	IB1	G3	23.8	Negative	negative	negative	less than 1/2
29	50	negative	5	IB2	G3	22.4	Negative	negative	negative	less than 1/2
30	34	negative	5	IIA2	G2	22.1	Negative	negative	negative	more than 1/2
31	42	negative	0.5	IIA2	G3	21.8	Negative	negative	negative	less than 1/2
32	34	negative	5	IB1	G2	21.9	Negative	negative	positive	more than 1/2
33	41	negative	2.5	IA2	G2	20.9	Negative	negative	negative	less than 1/2
34	41	negative	1.5	IB1	G2	21.1	Negative	negative	negative	more than 1/2
35	43	positive	2	IB1	G1	26.7	positive	positive	negative	more than 1/2
36	42	negative	2	IB1	G2	23.4	Negative	negative	negative	more than 1/2
37	53	positive	5	IB2	G3	24.1	positive	positive	negative	less than 1/2
38	31	negative	5	IB1	G3	24.5	Negative	negative	negative	less than 1/2
39	47	negative	1.5	IB1	G3	23.5	Negative	negative	negative	more than 1/2
40	36	negative	1.5	IIA1	G3	28.1	Negative	negative	positive	more than 1/2

41	42	negative	1.5	IB1	G3	26.7	Negative	negative	negative	more than 1/2
42	53	negative	4.5	IA2	G2	21.3	Negative	negative	negative	more than 1/2
43	50	negative	4.5	IB1	G3	24.1	Negative	negative	negative	less than 1/2
44	42	negative	0.5	IIA1	G3	26.1	Negative	negative	negative	less than 1/2
45	38	positive	5.5	IB1	G3	23.5	positive	negative	negative	more than 1/2
46	40	negative	0.5	IB2	G3	28	Negative	negative	negative	less than 1/2
47	42	negative	4.5	IB1	G2	21.9	Negative	negative	positive	more than 1/2
48	37	positive	3	IIA1	G3	23.3	positive	negative	negative	more than 1/2
49	42	positive	2	IIA1	G2	28.1	positive	negative	negative	more than 1/2
50	39	positive	5	IB1	G3	29.1	positive	negative	negative	more than 1/2
51	49	negative	2	IB2	G3	27.3	Negative	positive	positive	less than 1/2
52	45	negative	3	IB1	G2	22.8	Negative	negative	negative	less than 1/2
53	49	negative	2.5	IB1	G3	26.9	Negative	negative	negative	more than 1/2
54	43	negative	2.5	IB1	G3	22.9	Negative	negative	negative	more than 1/2
55	29	negative	3	IIA2	G3	22.5	Negative	negative	negative	less than 1/2
56	30	negative	3	IB2	G2	23.8	positive	negative	positive	more than 1/2
57	40	negative	2.5	IB2	G2	22.6	Negative	negative	negative	more than 1/2
58	38	negative	0.5	IIA2	G3	22.1	Negative	negative	negative	less than 1/2
59	39	negative	2	IB2	G3	21.8	Negative	negative	positive	less than 1/2
60	40	negative	1.5	IB1	G2	21.9	Negative	negative	negative	more than 1/2
61	32	negative	2	IB1	G3	28.9	Negative	negative	positive	more than 1/2
62	54	positive	2	IB2	G2	28.5	positive	negative	negative	less than 1/2
63	49	negative	2	IB2	G2	21.5	Negative	negative	negative	more than 1/2
64	43	negative	4.5	IB1	G3	21.3	Negative	positive	negative	less than 1/2
65	40	positive	3.5	IB1	G2	23.4	positive	negative	negative	more than 1/2
66	53	negative	3	IB1	G3	29.9	positive	negative	negative	more than 1/2
67	40	negative	2.5	IB1	G3	24.5	Negative	negative	negative	less than 1/2
68	56	negative	4	IB1	G3	22.3	Negative	negative	negative	more than 1/2
69	51	negative	1	IB2	G3	24.1	Negative	negative	negative	more than 1/2
70	29	negative	3	IIA1	G3	23.5	Negative	negative	positive	less than 1/2
71	42	negative	2.5	IB1	G2	21.9	Negative	negative	positive	less than 1/2
72	60	negative	4.5	IIA1	G3	23.3	Negative	positive	negative	more than 1/2
73	60	negative	2.5	IIA2	G2	31.2	Negative	negative	negative	more than 1/2
74	44	negative	5	IIA2	G3	30.3	positive	negative	negative	more than 1/2
75	38	negative	3	IB2	G3	22.8	Negative	negative	positive	more than 1/2
76	42	negative	0.5	IB1	G3	22.9	Negative	negative	negative	less than 1/2
77	43	positive	3.5	IB2	G1	28.9	positive	negative	negative	more than 1/2
78	32	negative	2.5	IIA2	G3	22.5	Negative	negative	negative	more than 1/2
79	49	negative	3	IIA1	G2	23.7	Negative	negative	negative	more than 1/2
80	37	negative	0.5	IB1	G2	22.6	Negative	negative	negative	less than 1/2
81	40	negative	2.5	IA2	G3	27.8	Negative	negative	negative	less than 1/2
82	35	positive	3	IB1	G3	21.1	Negative	negative	negative	less than 1/2
83	45	negative	1.5	IB1	G2	21.8	Negative	negative	negative	less than 1/2
84	32	negative	2	IA2	G3	21.9	Negative	negative	negative	more than 1/2

85	49	negative	2.5	IA2	G2	19.5	Negative	negative	negative	more than 1/2
86	36	positive	5	IB2	G3	21.3	Negative	negative	negative	more than 1/2
87	62	negative	2.5	IIA2	G2	23.4	Negative	positive	negative	more than 1/2
88	41	negative	3.5	IIA1	G3	22.9	Negative	negative	positive	more than 1/2
89	46	negative	3.5	IIA1	G3	22.5	Negative	negative	negative	less than 1/2
90	42	negative	3	IB2	G2	21.1	Negative	negative	negative	less than 1/2
91	36	negative	3	IB1	G2	22.9	Negative	negative	negative	less than 1/2
92	45	negative	3	IB1	G2	22.1	Negative	negative	negative	more than 1/2
93	30	negative	3.5	IB1	G2	23.8	Negative	negative	negative	less than 1/2
94	42	negative	5	IB1	G2	26.7	Negative	negative	positive	more than 1/2
95	43	negative	2	IB1	G3	25.9	Negative	negative	negative	less than 1/2
96	28	negative	2	IIA1	G3	21.9	Negative	negative	negative	less than 1/2
97	55	positive	4.5	IA2	G2	21.5	positive	negative	negative	more than 1/2
98	51	negative	2.5	IA2	G3	28	Negative	negative	negative	more than 1/2
99	51	negative	2	IB1	G3	29.1	Negative	negative	negative	less than 1/2
100	30	negative	2.5	IB1	G3	21.3	Negative	negative	negative	more than 1/2
101	44	negative	2	IIA2	G2	23.4	Negative	negative	positive	more than 1/2
102	41	positive	4.5	IB1	G2	24.5	Negative	negative	negative	more than 1/2
103	45	positive	3	IB1	G1	22.3	Negative	negative	negative	more than 1/2
104	43	negative	3	IB1	G3	32	Negative	negative	positive	less than 1/2
105	45	negative	3	IB1	G3	24.1	Negative	negative	negative	more than 1/2
106	49	positive	2.5	IIA1	G2	24.5	Negative	negative	negative	more than 1/2
107	43	negative	3.5	IB2	G3	21.8	Negative	negative	negative	less than 1/2
108	34	negative	1.5	IB1	G3	31.5	Negative	negative	negative	more than 1/2
109	68	negative	5	IIA1	G3	32.2	Negative	negative	negative	more than 1/2
110	64	negative	3	IB1	G2	23.5	Negative	negative	negative	less than 1/2
111	51	negative	3	IA2	G3	22.8	Negative	negative	positive	less than 1/2
112	40	negative	0.5	IB1	G2	24.9	Negative	negative	negative	less than 1/2
113	34	negative	1.5	IB1	G3	22.5	Negative	negative	negative	less than 1/2
114	47	negative	4.5	IA2	G3	28.4	Negative	negative	negative	less than 1/2
116	34	negative	1.5	IB2	G3	24.7	Negative	negative	positive	more than 1/2
116	27	negative	5	IIA1	G3	26.7	Negative	negative	negative	more than 1/2
117	31	negative	4.5	IB1	G3	23.6	Negative	negative	negative	more than 1/2
118	42	negative	4.5	IB1	G2	24.3	Negative	negative	negative	less than 1/2
119	35	positive	1.5	IB2	G2	26.1	Negative	negative	negative	more than 1/2
120	38	negative	0.5	IB1	G1	20.5	Negative	negative	negative	less than 1/2
121	44	negative	2	IB1	G3	26.7	Negative	negative	negative	less than 1/2
122	50	negative	5	IB1	G3	21.9	Negative	negative	negative	less than 1/2
123	34	negative	2	IB1	G1	23.5	Negative	negative	negative	more than 1/2
124	42	negative	1.5	IIA2	G3	22.8	Negative	negative	negative	less than 1/2
125	45	negative	5	IIA1	G3	28.1	Negative	negative	negative	more than 1/2
126	41	negative	1.5	IB1	G2	22.1	Negative	negative	negative	less than 1/2
127	41	positive	1.5	IB1	G1	28	Negative	negative	negative	more than 1/2
128	43	positive	2.5	IB1	G2	22.5	positive	negative	negative	more than 1/2

129	42	negative	2	IB1	G3	28.1	Negative	negative	negative	more than 1/2
130	51	positive	2	IB1	G3	29.1	positive	positive	negative	less than 1/2
131	31	negative	2.5	IB1	G2	23.8	Negative	negative	negative	less than 1/2
132	47	negative	2	IB2	G3	22.6	Negative	negative	negative	more than 1/2
133	45	negative	1.5	IIA1	G3	25	Negative	negative	negative	less than 1/2
134	32	negative	2	IIA2	G3	21.8	Negative	negative	negative	more than 1/2
135	49	negative	2.5	IIA2	G2	23.9	Negative	negative	negative	more than 1/2
136	39	positive	3	IB1	G3	27.3	Negative	negative	negative	more than 1/2
137	62	negative	2.5	IB1	G2	28.9	Negative	negative	negative	more than 1/2
138	41	negative	2	IIA2	G2	28.9	Negative	positive	positive	more than 1/2
139	46	negative	3.5	IB1	G2	19.1	Negative	negative	negative	less than 1/2
140	42	negative	3	IIA1	G3	29.5	Negative	negative	negative	less than 1/2
141	36	negative	4.5	IB1	G2	21.3	Negative	negative	negative	less than 1/2
142	29	negative	3	IB1	G3	23.4	Negative	negative	negative	more than 1/2
143	45	negative	3.5	IB1	G3	29.9	Negative	negative	negative	less than 1/2
144	42	negative	3	IIA1	G3	31.2	Negative	negative	positive	more than 1/2
145	43	negative	4.5	IB2	G3	21.9	Negative	negative	negative	less than 1/2
146	28	negative	2	IB1	G3	31.3	Negative	negative	negative	less than 1/2
147	55	positive	3	IB1	G2	28.9	positive	negative	negative	more than 1/2
148	51	negative	4.5	IB1	G3	22.5	Negative	negative	negative	more than 1/2
149	51	negative	2	IB1	G3	27.8	Negative	negative	negative	less than 1/2
150	30	negative	2.5	IB1	G3	28.7	Negative	negative	negative	more than 1/2
151	44	negative	2	IIA1	G3	25.9	Negative	negative	negative	more than 1/2
152	41	positive	4.5	IIA1	G2	31.3	Negative	negative	negative	more than 1/2
153	50	positive	3	IB1	G3	28.9	Negative	negative	negative	more than 1/2
154	43	negative	3	IB2	G2	21.1	Negative	negative	positive	less than 1/2
155	35	negative	3	IB1	G3	22.9	Negative	negative	positive	more than 1/2
156	49	positive	5.5	IB1	G3	28.8	Negative	negative	negative	more than 1/2
157	43	negative	3.5	IB1	G3	22.1	Negative	negative	negative	less than 1/2
158	34	negative	1.5	IB1	G3	23.8	Negative	negative	negative	more than 1/2
159	68	negative	5	IB1	G3	25	Negative	negative	negative	less than 1/2
160	64	negative	3	IB1	G3	26.7	Negative	negative	negative	less than 1/2
161	51	negative	0.5	IIA1	G2	21.5	Negative	negative	positive	less than 1/2
162	40	negative	0.5	IB1	G2	25	Negative	negative	negative	less than 1/2
163	34	negative	1.5	IB1	G3	21.8	Negative	negative	negative	less than 1/2
164	47	negative	4.5	IA2	G3	23.9	Negative	negative	negative	less than 1/2
165	27	negative	1	IB1	G2	26.9	Negative	negative	negative	more than 1/2
166	34	negative	5	IB1	G3	22.1	Negative	negative	negative	more than 1/2
167	46	negative	3.5	IB1	G2	23.3	Negative	negative	negative	more than 1/2
168	47	negative	4.5	IB1	G2	23.4	Negative	negative	negative	less than 1/2
169	27	negative	2	IB2	G3	21.9	Negative	negative	positive	more than 1/2
170	34	negative	5	IB1	G2	23.5	Negative	negative	negative	more than 1/2
171	46	negative	4.5	IIA2	G3	28	Negative	negative	negative	more than 1/2
172	42	negative	4.5	IB1	G3	29.1	Negative	negative	negative	less than 1/2

173	35	negative	5	IB1	G3	21.1	Negative	negative	negative	more than 1/2
174	38	negative	5	IB2	G3	32	Negative	negative	negative	less than 1/2
175	34	negative	4.5	IIA2	G3	22.9	Negative	negative	negative	less than 1/2
176	50	negative	3.5	IA2	G2	32.5	Negative	negative	negative	less than 1/2
177	34	negative	5	IB1	G3	24.1	Negative	negative	positive	more than 1/2
178	42	negative	0.5	IB2	G2	22.8	Negative	negative	negative	less than 1/2
179	45	negative	5	IB1	G3	22.5	Negative	negative	negative	less than 1/2
180	41	negative	2	IB1	G3	32	Negative	positive	negative	less than 1/2
181	41	positive	1.5	IB1	G3	29.4	Negative	negative	negative	less than 1/2
182	43	positive	3	IB2	G3	26.7	positive	negative	negative	more than 1/2
183	42	negative	2	IIA2	G3	24.7	Negative	negative	negative	more than 1/2
184	51	positive	5	IB1	G2	28.1	positive	negative	negative	less than 1/2
185	31	negative	4.5	IB1	G2	27.7	Negative	negative	negative	less than 1/2
186	47	negative	5	IB2	G3	23.6	Negative	negative	negative	more than 1/2
187	36	negative	4.5	IB1	G3	24.3	Negative	negative	positive	more than 1/2
188	42	negative	1	IB1	G2	22.5	Negative	negative	negative	more than 1/2
189	53	negative	2	IB1	G3	26.1	Negative	negative	negative	more than 1/2
190	50	negative	1.5	IIA1	G2	21.9	Negative	negative	negative	less than 1/2
191	42	negative	0.5	IB1	G3	23.5	Negative	negative	negative	less than 1/2
192	38	positive	2	IB1	G2	28	positive	negative	negative	more than 1/2
193	40	negative	0.5	IB1	G3	22.8	Negative	negative	negative	less than 1/2
194	42	negative	4.5	IB2	G3	22.1	Negative	negative	negative	more than 1/2
195	37	positive	3	IB1	G2	28.1	positive	negative	negative	less than 1/2
196	42	positive	2.5	IIA2	G2	29.9	Negative	negative	negative	more than 1/2
197	39	positive	2.5	IIA1	G3	32.2	positive	negative	negative	more than 1/2
198	49	negative	5	IB1	G2	23.5	Negative	negative	negative	less than 1/2
199	45	negative	3	IB2	G2	30.9	Negative	negative	positive	less than 1/2
200	49	negative	2	IB1	G3	28.9	Negative	positive	negative	more than 1/2
201	43	negative	2.5	IB1	G3	23.8	Negative	negative	negative	more than 1/2
202	53	negative	3	IB1	G3	27.8	Negative	negative	negative	less than 1/2
203	42	negative	4.5	IB1	G1	26.9	positive	negative	negative	more than 1/2
204	40	negative	2	IB1	G3	22.6	Negative	negative	negative	more than 1/2
205	39	negative	4.5	IIA2	G2	24.1	Negative	negative	negative	less than 1/2
206	37	negative	2	IIA2	G2	21.8	Negative	negative	positive	less than 1/2
207	42	positive	4.5	IB1	G2	25.9	Negative	negative	negative	more than 1/2
208	43	positive	2	IIA1	G3	30.6	positive	positive	negative	more than 1/2
209	44	negative	2.5	IB1	G3	23.9	Negative	negative	negative	more than 1/2
210	68	positive	1.5	IB1	G3	28.9	positive	positive	negative	less than 1/2
211	31	negative	0.5	IB1	G3	21.1	Negative	negative	negative	less than 1/2
212	47	negative	2	IB2	G2	21.3	Negative	negative	negative	more than 1/2
213	36	negative	2	IB2	G3	27.8	Negative	negative	positive	more than 1/2
214	43	negative	2	IB1	G3	26.9	Negative	negative	negative	more than 1/2
215	55	negative	4.5	IB1	G3	23.4	Negative	negative	negative	more than 1/2
216	50	negative	1	IIA1	G3	25.8	Negative	negative	negative	less than 1/2

217	41	negative	0.5	IB1	G3	28.9	Negative	negative	negative	less than 1/2
218	38	negative	3	IB1	G3	24.9	positive	negative	negative	more than 1/2
219	40	negative	0.5	IB2	G3	29.8	Negative	negative	negative	less than 1/2
220	42	negative	3.5	IB1	G2	22.5	Negative	negative	negative	more than 1/2
221	37	positive	3.5	IIA1	G2	31.4	Negative	negative	negative	more than 1/2
222	42	positive	3.5	IA2	G2	28.7	positive	negative	negative	more than 1/2
223	40	positive	2.5	IA2	G2	25.9	positive	negative	negative	more than 1/2
224	49	negative	2	IA2	G3	21.1	Negative	positive	positive	less than 1/2
225	44	negative	3.5	IA2	G3	22.9	Negative	negative	negative	less than 1/2
226	49	negative	1.5	IIA1	G3	22.1	Negative	negative	negative	more than 1/2
227	43	negative	2.5	IB1	G3	21.8	Negative	negative	positive	more than 1/2
228	51	negative	3	IB1	G3	20.5	Negative	negative	negative	less than 1/2
229	31	negative	3.5	IB1	G2	31.3	Negative	negative	positive	more than 1/2
230	40	negative	4	IB1	G2	28.9	Negative	negative	negative	more than 1/2
231	38	negative	0.5	IIA2	G3	21.9	Negative	negative	negative	less than 1/2
232	39	negative	1.5	IB2	G3	23.5	Negative	negative	positive	less than 1/2
233	44	negative	3.5	IB1	G2	29.8	Negative	negative	negative	more than 1/2
234	32	negative	2	IIA1	G3	22.8	Negative	negative	negative	more than 1/2
235	36	negative	4.5	IB2	G3	26.7	Negative	negative	positive	more than 1/2
236	42	negative	4.5	IB2	G2	28.9	Negative	negative	negative	more than 1/2
237	55	negative	4	IB2	G3	22.1	Negative	negative	negative	more than 1/2
238	50	negative	1.5	IB1	G2	22.5	Negative	negative	negative	less than 1/2
239	42	negative	2	IIA2	G3	23.8	Negative	negative	negative	less than 1/2
240	37	positive	5	IIA1	G2	28	positive	negative	negative	more than 1/2
241	40	negative	1	IB1	G1	22.7	Negative	negative	negative	less than 1/2
242	42	negative	2	IB1	G3	23.8	Negative	negative	negative	more than 1/2
243	37	positive	3	IB2	G2	28.1	positive	negative	negative	less than 1/2
244	41	positive	5	IIA2	G3	21.8	Negative	negative	negative	more than 1/2
245	39	positive	3.5	IB2	G2	30.5	positive	negative	negative	more than 1/2
246	49	negative	5	IIA2	G2	23.9	Negative	negative	negative	less than 1/2
247	41	negative	3	IB2	G2	19.1	Negative	negative	negative	less than 1/2
248	39	positive	4.5	IIA2	G2	32.5	positive	negative	negative	less than 1/2
249	32	negative	4.5	IA2	G3	25.6	Negative	negative	positive	less than 1/2
250	37	negative	0.5	IB2	G3	29.9	Negative	negative	negative	less than 1/2
251	51	negative	1.5	IIA1	G3	26.7	Negative	negative	negative	less than 1/2
252	40	negative	0.5	IIA2	G2	28.7	Negative	negative	negative	less than 1/2
253	38	negative	1.5	IB2	G3	21.3	Negative	negative	negative	less than 1/2
254	47	negative	4.5	IB1	G3	23.7	Negative	negative	positive	less than 1/2
255	28	negative	1.5	IA2	G2	21.5	Negative	negative	negative	more than 1/2
256	34	negative	1.5	IB1	G3	22.5	Negative	negative	negative	more than 1/2
257	46	negative	2	IA2	G2	21.8	Negative	negative	negative	more than 1/2
258	47	negative	4	IIA1	G2	27.7	Negative	negative	positive	less than 1/2
259	30	negative	4.5	IIA1	G3	26.9	Negative	negative	negative	more than 1/2
260	34	negative	4.5	IB2	G2	22.1	Negative	negative	negative	more than 1/2

FIGO, the International Federation of Gynecology and Obstetrics; LVSI, lymphovascular space invasion; LNM, lymph node metastasis; BMI, body mass index; IHC, immunohistochemistry.

Table S2. Associations of FABP5 expression levels with clinicopathological factors in early-stage cervical cancer.

	N	FABP5		*P*
	260	Low (0-4)[a] 169	High (5-6)[a] 91	
Age (years)				0.301
≤ 35	55	39	16	
> 35	205	130	75	
BMI (kg/m^2)				0.013
≤ 25	158	112	46	
> 25	102	57	45	
FIGO stage				0.001
Ia2	21	21	0	
Ib1	127	82	45	
Ib2	46	31	15	
IIa1	38	25	13	
IIa2	28	10	18	
Tumour size (cm)				0.02
≤ 4	186	129	57	
> 4	74	40	34	
LNM				< 0.001
Positive	49	14	35	
Negative	211	155	56	
LVSI				0.004
Positive	33	14	19	
Negative	227	155	72	
Differentiation				0.541
G1	17	9	8	
G2	91	59	32	
G3	152	101	51	
Stomal invasion				0.081
≤ 1/2	122	86	36	
> 1/2	138	83	55	
Parametrial invasion				0.194
Positive	16	8	8	
Negative	244	161	83	
Vaginal invasion				0.072
Positive	40	21	19	
Negative	220	148	72	

FIGO, the International Federation of Gynecology and Obstetrics; LVSI, lymphovascular space invasion; LNM, lymph node metastasis; BMI, body mass index.

[a] The immunostaining scores of FABP5

Table S3. Multivariate Cox's proportional hazards model analysis of relapse-free and overall survivals.

Variables	Overall survival		Relapse-free probability	
	HR (95% CI)	*P*	HR (95% CI)	*P*
Age (year)	1.083 (1.049-1.118)	< 0.001	1.075 (1.055-1.124)	< 0.001
Tumor size (cm)	1.579 (1.264-1.973)	< 0.001	1.552 (1.245-1.935)	< 0.001
LNM		< 0.001		< 0.001
Negative (reference)	1		1	
Positive	6.698 (3.649-12.294)		5.996 (3.261-11.027)	
FABP5		0.004		0.004
Low (reference)	1		1	
High	2.517 (1.334-4.750)		2.556 (1.345-4.857)	

HR, hazard ratio; 95% CI, 95% confidence interval; LNM, lymph node metastasis.
For the stepwise multivariate analysis, forward LR method was used to select significant variables.
Variables entered for analysis were the following: age, FIGO stage, tumour size, LNM, BMI, LVSI, differentiation, stromal invasion, vaginal invasion, parametrial invasion, FABP5.

Table S4. Primers and siRNAs used in the study

Gene	Sequences
FABP5	Forward 5'-GAATACATGAAGGAGCTAGG-3'<br>Reverse 5'- ACTGAGCTTGGTCATTCTC-3'
FASN	Forward 5'- AAGGACCTGTCTAGGTTTGATGC-3'<br>Reverse 5'- TGGCTTCATAGGTGACTTCCA-3'
ACC1	Forward 5'- AGGAAGATGGTGTCCGCTCTG-3'<br>Reverse 5'- GGGGAGATGTGCTGGGTCAT-3'
CPT1A	Forward 5'- TCCAGTTGGCTTATCGTGGTG-3'<br>Reverse 5'- TCCAGAGTCCGATTGATTTTTGC-3'
ACOX1	Forward 5'- ACTCGCAGCCAGCGTTATG-3'<br>Reverse 5'- AGGGTCAGCGATGCCAAAC-3'
HSL	Forward 5'-TCAGTGTCTAGGTCAGACTGG-3'<br>Reverse 5'- AGGCTTCTGTTGGGTATTGGA-3''
GAPDH	Forward 5'-ATCACCATCTTCCAGGAGCGA-3'<br>Reverse 5'-CCTTCTCCATGGTGGTGAAGAC-3'
TWIST1	Forward 5'-GTCCGCAGTCTTACGAGGAG-3'<br>Reverse 5'-GCTTGAGGGTCTGAATCTTGCT-3'
MMP9	Forward 5'-TGTACCGCTATGGTTACACTCG-3'<br>Reverse 5'-GGCAGGGACAGTTGCTTCT-3'
VEGF-C	Forward 5'-GAGGAGCAGTTACGGTCTGTG-3'<br>Reverse 5'-TCCTTTCCTTAGCTGACACTTGT-3'
miR-144-3p	Forward 5'-CCCTACAGTATAGATGATG-3'<br>Reverse 5'-TGCAGGGTCCGAGGT-3'
U6	Forward 5'-ACAGATCTGTCGGTGTGGCAC-3'<br>Reverse 5'-GGCCCCGGATTATCCGACATTC-3'
siFABP5	Sense 5'-UGUACCCUGGGAGAGAAGUTT-3'<br>Antisense 5'-ACUUCUCUCCCAGGGUACATT-3'
siNC	Sense 5'-UUCUCCGAACGUGUCACGUTT-3'<br>Antisense 5'-ACGUGACACGUUCGGAGAATT-3'
shFABP5	5'-TGGGAAGGAAAGCACAATA-3'
shNC	5'-TTCTCCGAACGTGTCACGT-3'

Table S5. Antibodies used in the study.

Antibody	Catalog#	Working concentration	Manufacturer
FABP5	39926S	1:1000	CST
CPT1A	12252S	1:1000	CST
ACC1	3676S	1:1000	CST
ACOX1	ab184032	1:1000	Abcam
FASN	ab22759	1:1000	Abcam
GAPDH	2118S	1:500	CST
VEGF-C	sc-374628	1:1000	Santa Cruz
E-Cadherin	20874-1-AP	1:5000	Proteintech
N-Cadherin	22018-1-AP	1:5000	Proteintech
Vimentin	5741S	1:1000	CST
CA9	ab15086	1:1000	Abcam
HSL	sc-74489	1:800	Santa Cruz
p65	sc-8008	1:500	Santa Cruz
p84	ab487	1:800	Abcam

Supplementary figure

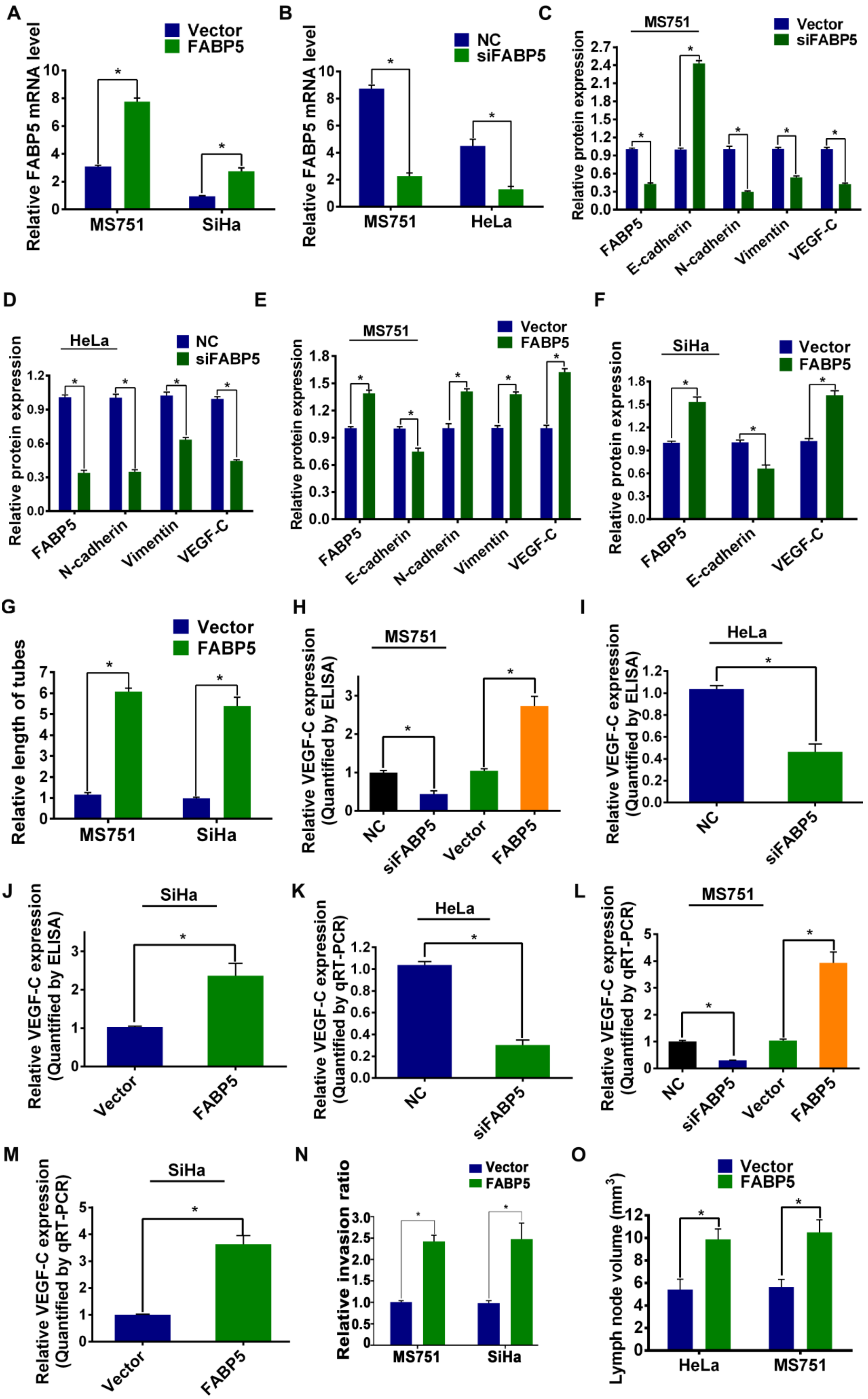
A
Vector
FABP5
Relative FABP5 mRNA level
MS751
SiHa
B
NC
siFABP5
Relative FABP5 mRNA level
MS751
HeLa
C
MS751
Vector
siFABP5
Relative protein expression
FABP5
E-cadherin
N-cadherin
Vimentin
VEGF-C
D
HeLa
NC
siFABP5
Relative protein expression
FABP5
N-cadherin
Vimentin
VEGF-C
E
MS751
Vector
FABP5
Relative protein expression
FABP5
E-cadherin
N-cadherin
Vimentin
VEGF-C
F
SiHa
Vector
FABP5
Relative protein expression
FABP5
E-cadherin
VEGF-C
G
Vector
FABP5
Relative length of tubes
MS751
SiHa
H
MS751
Relative VEGF-C expression (Quantified by ELISA)
NC
siFABP5
Vector
FABP5
I
HeLa
Relative VEGF-C expression (Quantified by ELISA)
NC
siFABP5
J
SiHa
Relative VEGF-C expression (Quantified by ELISA)
Vector
FABP5
K
HeLa
Relative VEGF-C expression (Quantified by qRT-PCR)
NC
siFABP5
L
MS751
Relative VEGF-C expression (Quantified by qRT-PCR)
NC
siFABP5
Vector
FABP5
M
SiHa
Relative VEGF-C expression (Quantified by qRT-PCR)
Vector
FABP5
N
Vector
FABP5
Relative invasion ratio
MS751
SiHa
O
Vector
FABP5
Lymph node volume (mm3)
HeLa
MS751

Figure S1. FABP5 promotes EMT, tube formation of HLECs, and the expression of VEGF-C in CCa. (A) Real-time PCR results showed the efficiencies of FABP5 overexpression in indicated cells. (B) Real-time PCR results showed the efficiencies of FABP5 knockdown in indicated cells. (C-F) Quantification of western blot analysis of EMT markers and VEGF-C in indicated cells with knockdown or overexpression of FABP5. (G) The effects of FABP5 on the tube formation of HLECs. (H-J) The effect of FABP5 on VEGF-C secretion was evaluated by ELISA. (K-M) The effect of FABP5 on VEGF-C expression was evaluated by qRT-PCR. (N) Relative invasion ratio of MS751 and SiHa cells with FABP5 overexpression. (O) Histogram analysis of the volume of lymph nodes of indicated cells. Error bars represent the mean ± S.D. of three independent experiments. *$P < 0.05$.

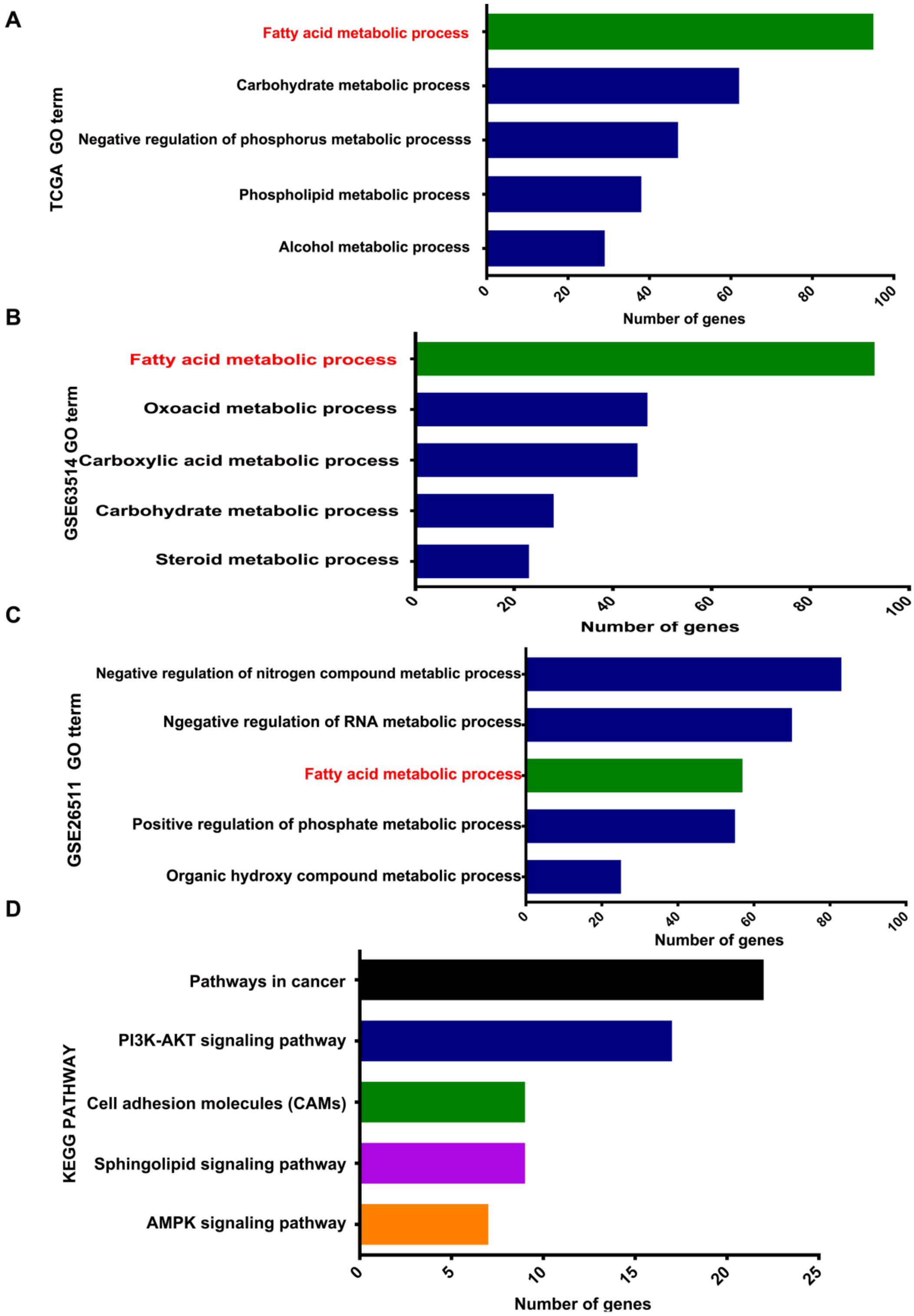

Figure S2. Bioinformatic analysis of genes correlated with FABP5. (A) The Gene Ontology analysis of genes correlated with FABP5 in TCGA dataset. (B) The Gene Ontology analysis of genes correlated with FABP5 in GSE63514 dataset. (C) The Gene Ontology analysis of genes correlated with FABP5 in GSE26511 dataset. (D) The KEGG pathway enrichment analysis of genes correlated with FABP5 co-existing in TCGA, GSE26511, GSE63514 datasets.

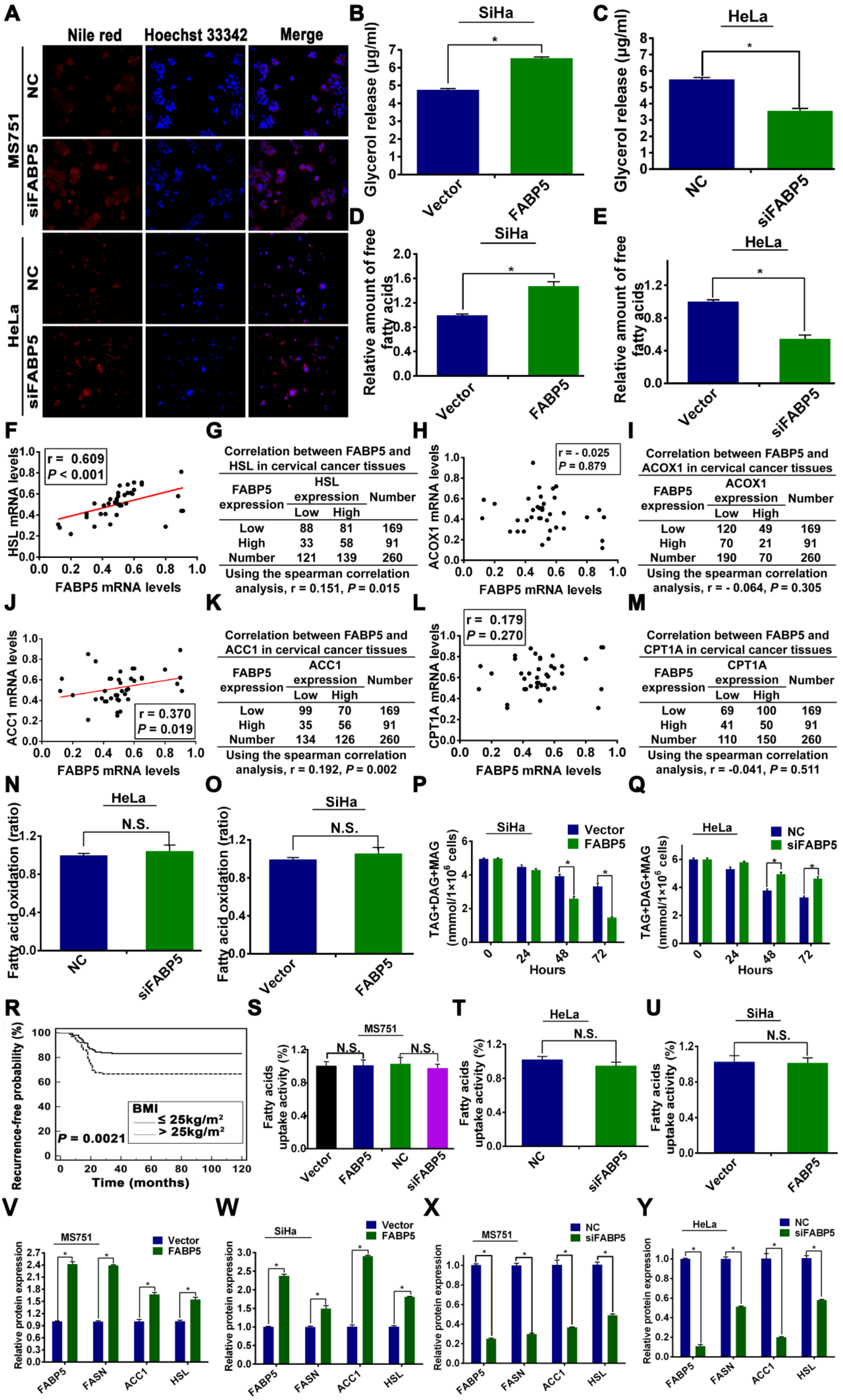
A
Nile red
Hoechst 33342
Merge
MS751
NC
siFABP5
HeLa
B
SiHa
Glycerol release (μg/ml)
Vector
FABP5
C
HeLa
NC
siFABP5
D
Relative amount of free fatty acids
E
F
r = 0.609
P < 0.001
HSL mRNA levels
FABP5 mRNA levels
G
Correlation between FABP5 and HSL in cervical cancer tissues
FABP5 expression | HSL expression Low | HSL expression High | Number
Low | 88 | 81 | 169
High | 33 | 58 | 91
Number | 121 | 139 | 260
Using the spearman correlation analysis, r = 0.151, P = 0.015
H
r = - 0.025
P = 0.879
ACOX1 mRNA levels
I
Correlation between FABP5 and ACOX1 in cervical cancer tissues
FABP5 expression | ACOX1 expression Low | ACOX1 expression High | Number
Low | 120 | 49 | 169
High | 70 | 21 | 91
Number | 190 | 70 | 260
Using the spearman correlation analysis, r = - 0.064, P = 0.305
J
ACC1 mRNA levels
r = 0.370
P = 0.019
K
Correlation between FABP5 and ACC1 in cervical cancer tissues
FABP5 expression | ACC1 expression Low | ACC1 expression High | Number
Low | 99 | 70 | 169
High | 35 | 56 | 91
Number | 134 | 126 | 260
Using the spearman correlation analysis, r = 0.192, P = 0.002
L
r = 0.179
P = 0.270
CPT1A mRNA levels
M
Correlation between FABP5 and CPT1A in cervical cancer tissues
FABP5 expression | CPT1A expression Low | CPT1A expression High | Number
Low | 69 | 100 | 169
High | 41 | 50 | 91
Number | 110 | 150 | 260
Using the spearman correlation analysis, r = -0.041, P = 0.511
N
HeLa
N.S.
Fatty acid oxidation (ratio)
O
SiHa
P
TAG+DAG+MAG (nmmol/1×10⁶ cells)
Hours
Q
R
Recurrence-free probability (%)
Time (months)
BMI
≤ 25kg/m²
> 25kg/m²
P = 0.0021
S
MS751
Fatty acids uptake activity (%)
T
U
V
Relative protein expression
FASN
ACC1
HSL
W
X
Y

Figure S3. Effects of FABP5 expression on fatty acid metabolism in CCa cells. (A) Lipid droplets were detected using Nile red in indicated cells with FABP5 knockdown. (B) Glycerol was measured in SiHa cells with FABP5 overexpression. (C) The amount of glycerol was measured in HeLa cells with FABP5 knockdown. (D) The amount of free fatty acids was measured in SiHa cells with FABP5 overexpression. (E) The amount of free fatty acids was measured in HeLa cells with FABP5 knockdown. (F, H, J, L) Scatter plot analysis of correlation between mRNA expression levels of FABP5 and HSL, ACOX1, ACC1, CPT1A in 40 cervical cancer tissues. (G, I, K, M) The relationships between the protein expression level of FABP5 and HSL, ACOX1, ACC1, CPT1A were examined based on IHC staining. (N) Fatty acid oxidation was measured in HeLa cells with FABP5 knockdown. (O) Fatty acid oxidation was measured in SiHa cells with FABP5 overexpression. (P) The amount of intracellular neutral lipids (TAG, DAG, and MAG) was measured in SiHa cells with FABP5 overexpression. (Q) The amount of intracellular neutral lipids (TAG, DAG, and MAG) was measured in HeLa cells with FABP5 knockdown. (R) Kaplan–Meier survival curves of RFS of patients with CCa according to BMI. (S-U) Effects of FABP5 on the fatty acids uptake activity in the indicated cells. (V-Y) Quantification of western blot analysis for protein levels of FASN, ACC1, HSL, and FABP5 in the indicated cells. Error bars represent the mean ± S.D. of three independent experiments. $^{*}P < 0.05$.

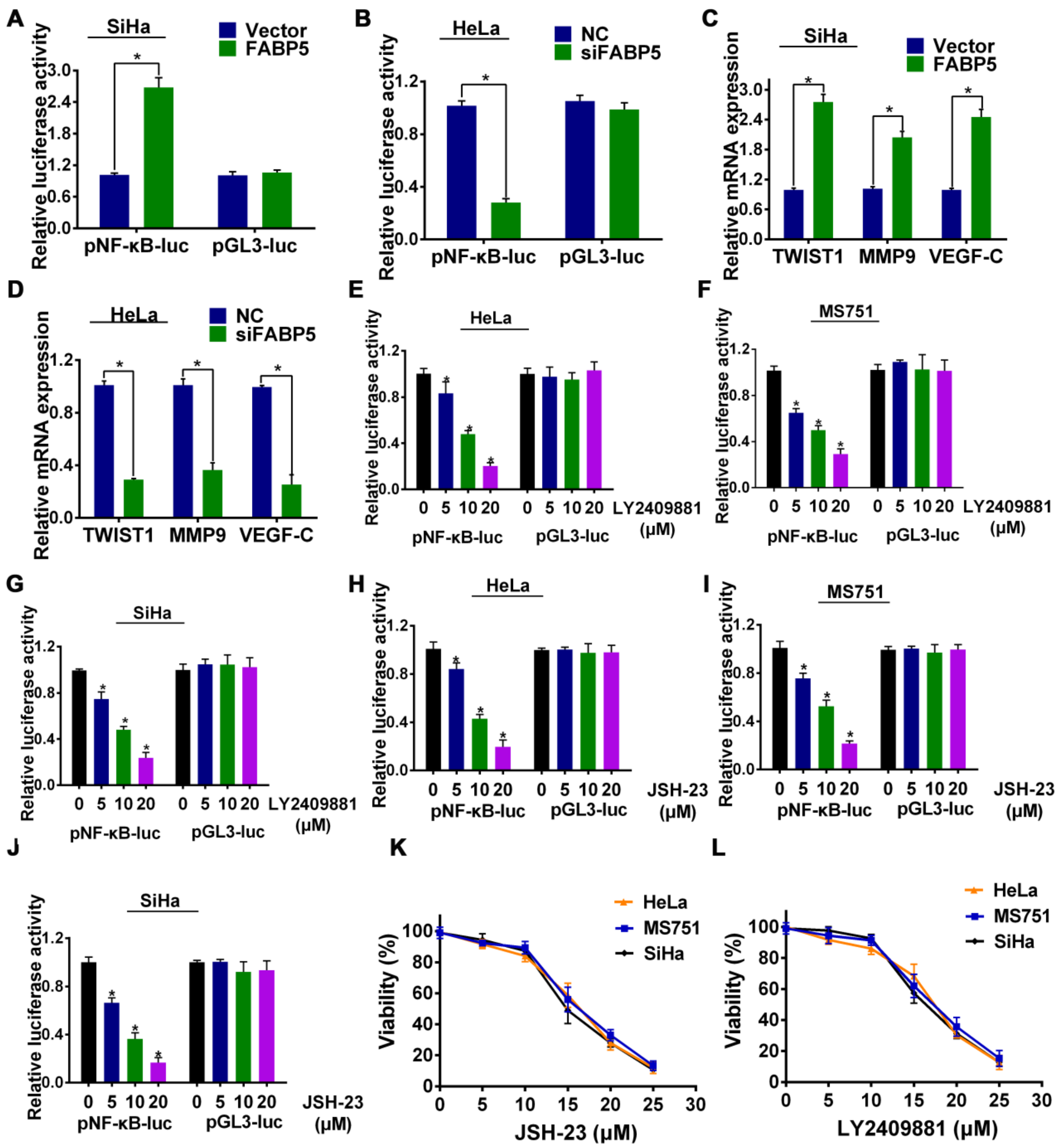

Figure S4. FABP5 activates NF-κB signaling pathway. (A, B) NF-κB transcriptional activity was assessed by luciferase reporter constructs in the indicated cells. (C, D) Real-time PCR analysis of TWIST1, MMP9 and VEGF-C in the indicated cells. (E-G) NF-κB signalling inhibitor LY2409881 inhibited the NF-κB transcriptional activity in a dose-dependent manner in the indicated cells. (H-J) NF-κB signalling inhibitor JSH-23 inhibited the NF-κB transcriptional activity in a dose-dependent manner in the indicated cells. (K, L) The effect of JSH-23 or LY2409881 on proliferation of CCa cells by CCK-8 assays. MS751, HeLa, SiHa cells were treated by JSH-23 or LY2409881 at the indicated concentrations for 48 hours. Error bars represent the mean ± S.D. of three independent experiments. *$P < 0.05$.

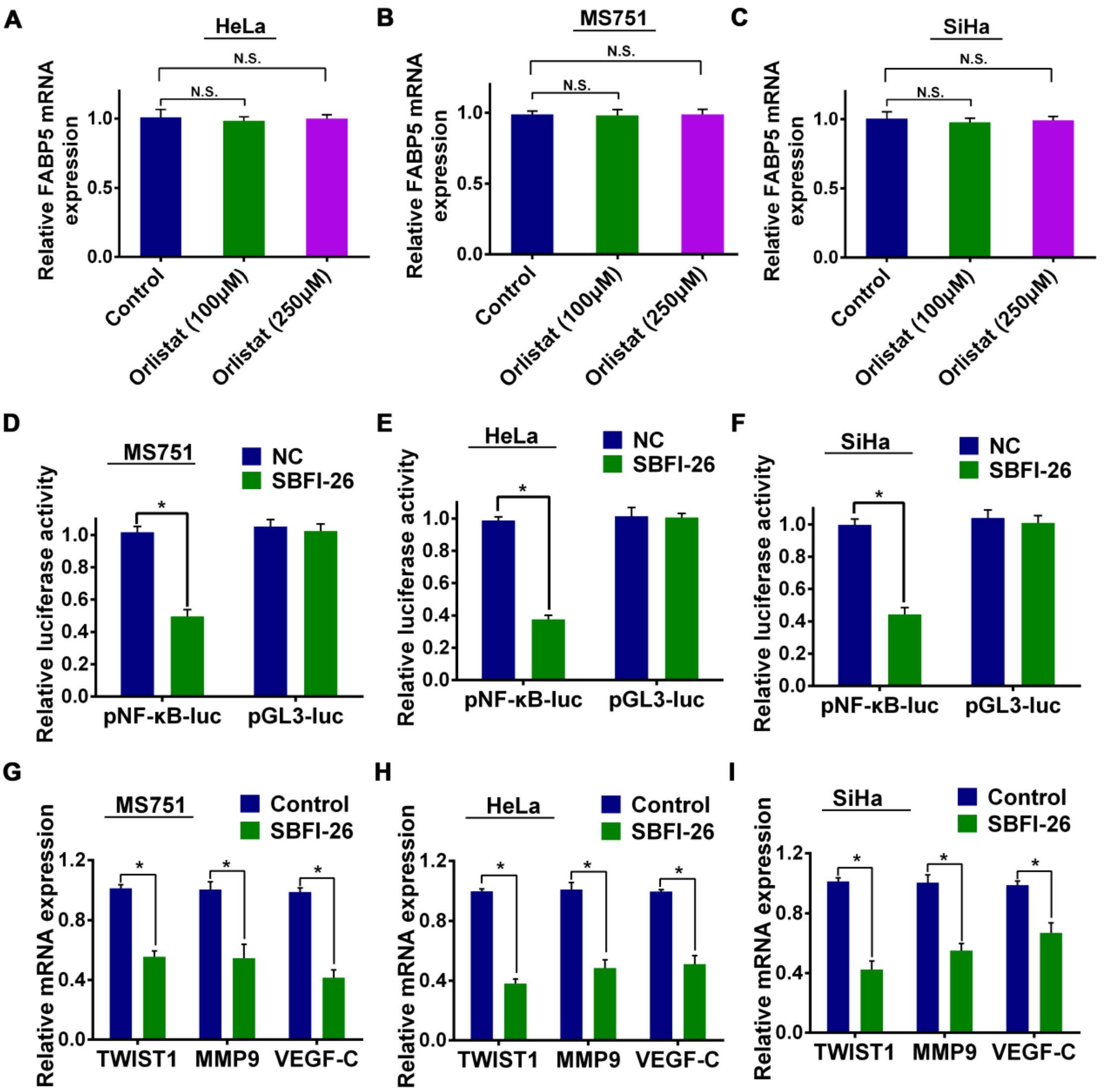

Figure S5. Effect of orlistat on the expression of FABP5 and the effect of SBFI-26 (FABP5 inhibitor) on the activity of NF-κB signaling in CCa cells. (A-C) The effect of orlistat on FABP5 mRNA expression in indicated cells. (D-F) NF-κB transcriptional activity was assessed by luciferase reporter constructs in the indicated cells treated by SBFI-26 (5 μM). (G-I) Real-time PCR analysis of NF-κB signaling downstream target genes (TWIST1, MMP9, and VEGF-C) in the indicated cells treated by SBFI-26 (5 μM).

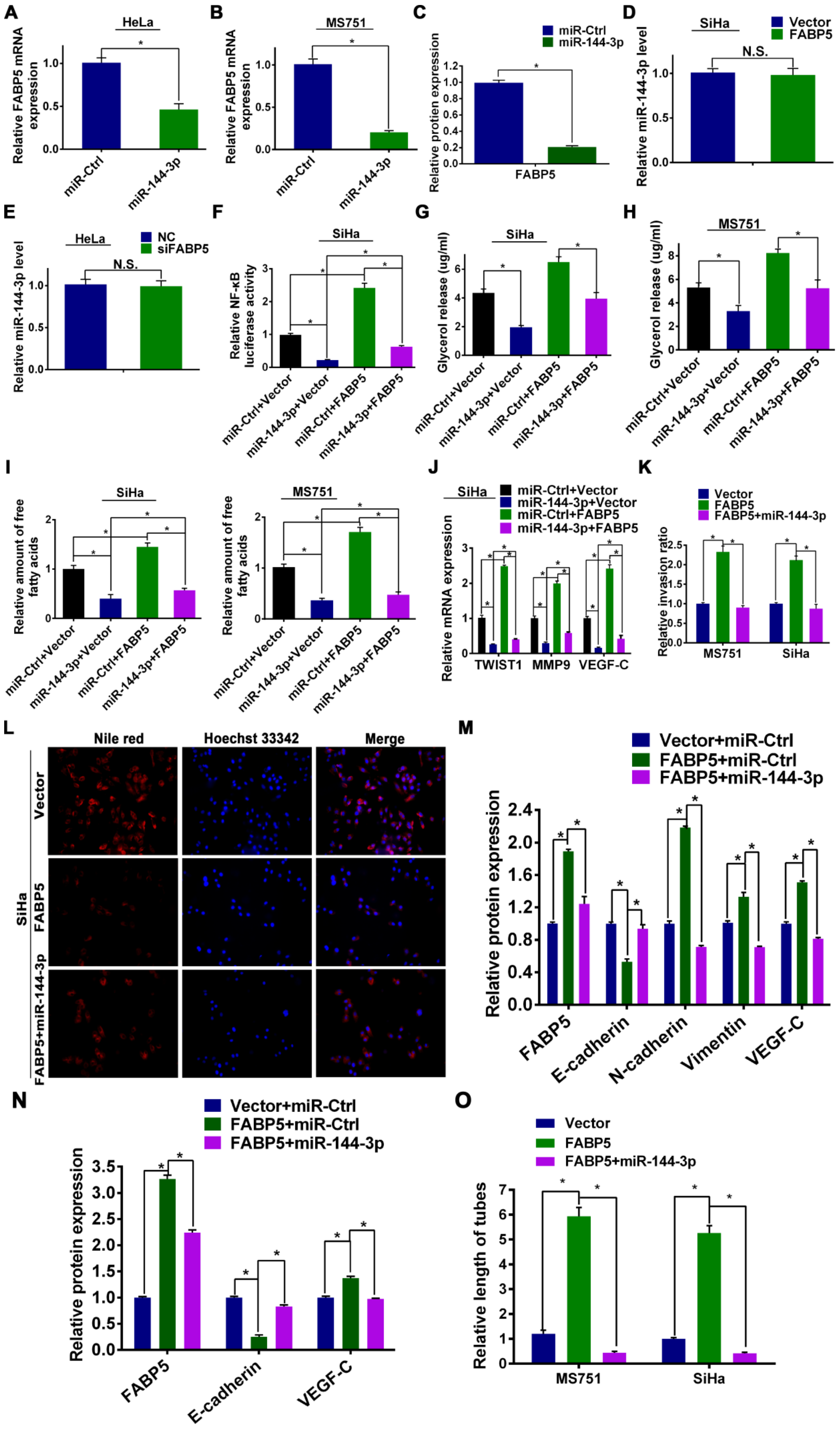
A
HeLa
Relative FABP5 mRNA expression
miR-Ctrl
miR-144-3p
B
MS751
C
miR-Ctrl
miR-144-3p
Relative protien expression
FABP5
D
SiHa
Vector
FABP5
N.S.
Relative miR-144-3p level
E
NC
siFABP5
F
Relative NF-κB luciferase activity
miR-Ctrl+Vector
miR-144-3p+Vector
miR-Ctrl+FABP5
miR-144-3p+FABP5
G
Glycerol release (ug/ml)
H
I
Relative amount of free fatty acids
J
Relative mRNA expression
TWIST1
MMP9
VEGF-C
K
Relative invasion ratio
FABP5+miR-144-3p
L
Nile red
Hoechst 33342
Merge
SiHa
M
Vector+miR-Ctrl
FABP5+miR-Ctrl
Relative protein expression
E-cadherin
N-cadherin
Vimentin
N
O
Relative length of tubes

Figure S6. Effect of miR-144-3p on FABP5 and tumor progression. (A, B) Effect of miR-144-3p mimic transfection on FABP5 expression levels in the indicated cells. (C) Quantification of western blot analysis for protein levels of FABP5 in MS751cells treated with miR-144-3p. (D, E) Effect of FABP5 on miR-144-3p expression levels in the indicated cells. (F) NF-κB transcriptional activity was assessed by luciferase reporter constructs in the indicated cells. (G, H) Effect of miR-144-3p mimic transfection on the amount of glycerol in the indicated cells. (I) Effect of miR-144-3p mimic transfection on the amount of free fatty acids in the indicated cells. (J) Real-time PCR analysis of NF-κB signaling downstream target genes (TWIST1, MMP9 and VEGF-C) in the indicated cells. (K) miR-144-3p attenuated the stimulatory effect of FABP5 on invasion ability in the indicated cells respectively. (L) Lipid drops were detected using Nile Red in SiHa cells treated with miR-144-3p mimics. (M, N) Quantification of western blot analysis of EMT markers and VEGF-C in indicated cells treated with miR-144-3p mimics. (O) miR-144-3p mimics attenuated the stimulatory effect of FABP5 on the tube formation of HLECs. Error bars represent the mean ± S.D. of three independent experiments. $^*P < 0.05$.

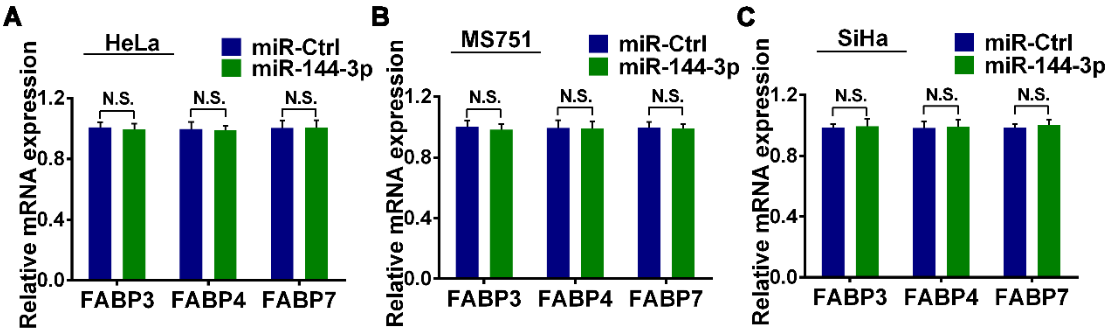

Figure S7. Effect of miR-144-3p on FABP3, FABP4, FABP7 and tumor progression. (A-C) Effect of miR-144-3p mimics transfection on FABP3, FABP4, and FABP7 expression levels in indicated cells.
